# The unequal adoption of ChatGPT exacerbates existing inequalities among workers

**DOI:** 10.1073/pnas.2414972121

**Published:** 2024-12-30

**Authors:** Anders Humlum, Emilie Vestergaard

**Affiliations:** ^a^Microeconomics Unit, Booth School of Business, University of Chicago, Chicago IL, 60637; ^b^Department of Economics, University of Copenhagen, Copenhagen 1353, Denmark

**Keywords:** technology adoption, labor productivity, ChatGPT, generative AI

## Abstract

We study the adoption of ChatGPT, the icon of Generative AI, using a large-scale survey linked to comprehensive register data in Denmark. Surveying 18,000 workers from 11 exposed occupations, we document that ChatGPT is widespread, especially among younger and less-experienced workers. However, substantial inequalities have emerged. Women are 16 percentage points less likely to have used the tool for work. Furthermore, despite its potential to lift workers with less expertise, users of ChatGPT earned slightly more already before its arrival, even given their lower tenure. Workers see a substantial productivity potential in ChatGPT but are often hindered by employer restrictions and a perceived need for training.

## Significance

Using a large-scale and representative survey, we examine who has adopted ChatGPT, how workers anticipate the technology will affect their jobs, and why some workers use it and others do not. We show how barriers to the adoption of ChatGPT have exacerbated some existing inequalities, with women and lower-earning workers less likely to use the tool. Younger and less experienced workers have adopted ChatGPT faster.

The arrival of ChatGPT marks the era of Generative AI, in which several high-skilled occupations may be disrupted (1). This paper studies who has adopted ChatGPT, how workers anticipate it will affect their jobs, and why some workers use it, and others do not.

In collaboration with Statistics Denmark, we surveyed a representative sample of 18,000 workers from 11 exposed occupations between November 2023 and January 2024. We link the survey responses to register data on individual labor market histories, earnings, wealth, education, and demographics to characterize heterogeneity in the adoption of ChatGPT.

We first document that ChatGPT is widespread in the exposed occupations: 41% of workers have used the tool for work, with adoption rates ranging from 65% for marketing professionals to 12% for financial advisors, and almost everyone is aware of it. The widespread adoption of ChatGPT, only a year after its first launch, solidifies it as a landmark event in technology history.

Second, we look within the exposed occupations and ask what characterizes workers who use ChatGPT. Existing evidence highlights workers with less prior expertise have the most to gain from ChatGPT and other Generative AI (2, 3), suggesting that the technology could help alleviate existing inequalities between workers (4). Consistent with this view, we find younger and less experienced workers are more likely to use ChatGPT. In particular, every year of age and experience is associated with a 0.7 and 0.6 percentage point lower likelihood of having used ChatGPT for work. However, despite the lower tenure, workers who use ChatGPT earned slightly more already before its arrival, reflecting that higher-achieving individuals within cohorts are more likely to use ChatGPT. Finally, we document a staggering gender gap in the adoption of ChatGPT: women are 16 percentage points less likely to have used ChatGPT for work than men in the same occupation. The gender gap is pervasive in all occupations, exists in various adoption measures, and persists when comparing coworkers within the same workplace handling the same types of job tasks.

Next, we examine how workers anticipate ChatGPT will impact their work activities. Workers in the exposed occupations see a substantial productivity potential in ChatGPT, estimating it can halve working times in about a third of their job tasks. Workers are twice as likely to state ChatGPT provides smaller rather than larger time savings for workers with greater expertise, consistent with existing evidence that the technology substitutes for human expertise (2). Workers expect little substitution between tasks in response to ChatGPT, with about 40% reporting they will not perform more of the tasks ChatGPT saves time completing.

Finally, we document that workers’ perceived time savings from ChatGPT are only a weak predictor of their use of the tool. For example, among workers who believe ChatGPT can halve the time to complete a task, only about 23% plan to use it within the next 2 wk. Workers report employer restrictions on use and needing training as the primary barriers to adoption. By randomly informing workers about the time savings from ChatGPT and observing no shifts in usage behavior in response, we confirm that these or other frictions are causally hindering workers from capitalizing on the perceived productivity gains of ChatGPT.

## 1. Materials and Methods

ChatGPT is the clear leader among generative AI chatbots, capturing 80 to 90% of total website traffic; see *SI Appendix*, section 2.A for details.

The data infrastructure in Denmark offers an ideal setting to study the adoption of ChatGPT. In particular, every Dane has a digital mailbox that Statistics Denmark can use to send survey invitations. We link the survey to the administrative registers at Statistics Denmark, which offers two advantages to this study. First, we observe detailed occupational codes for all workers, allowing us to target the survey to individuals in exposed occupations. Second, the registers contain a wealth of information about individuals, allowing us to study heterogeneity by workers’ labor market histories, earnings, wealth, education, and demographics.

### 1.1. Occupations.

We identify occupations that are exposed to ChatGPT using the expert assessment of Eloundou et al. (1). We use their “Direct Exposure (E1)” measure, which asks whether access to ChatGPT can halve the time an average worker takes to complete a task at equal quality. *SI Appendix*, section 1.A.1 describes the measure, which we call “productivity” in this paper.

Eloundou et al. (1) use a combination of human assessments and GPT prompts to classify the time productivity of ChatGPT in the Detailed Work Activities (DWAs) in the O*NET database. We replicate the GPT ratings of Eloundou et al. (1), applying minor adjustments to classify the most detailed Job Duties in O*NET.^*^

We include all occupations that i) have at least one job task that is exposed to ChatGPT, ii) are captured by a well-defined set of ISCO codes, and iii) contain enough workers for statistical analysis; see *SI Appendix*, section 1.A.3 for details. The resulting list of occupations is accountants, customer support specialists, financial advisors, HR professionals, IT support specialists, journalists, legal professionals, marketing professionals, office clerks, software developers, and teachers.

We include six representative job tasks for each occupation in our survey. *SI Appendix*, section 1.A.4 details our selection algorithm, and *SI Appendix*, section 6 lists the resulting job tasks for each occupation.

### 1.2. Survey.

Our survey focuses on workers’ use of and beliefs about ChatGPT in their job tasks. The survey includes an experiment, informing workers about expert assessments of ChatGPT in their job tasks, and a follow-up to see whether treatment effects persist. *SI Appendix*, section 1.B describes the blocks of the survey, and *SI Appendix*, section 8 lists the full questionnaire.

#### 1.2.1. Sample.

We invited 100,000 workers from the 11 exposed occupations to participate in our survey between November 2023 and January 2024, distributing a follow-up 2 wk after workers’ initial responses. The invitation letter in *SI Appendix*, section 7 informed invitees about data processing and requested their consent before participation in the survey. We preregistered our survey and experiment at AEA-RCT-R-0012527 and received IRB approval from the University of Copenhagen.

The main survey achieved about 18,000 valid and complete responses, which we use in our main analysis. *SI Appendix*, section 1.C describes our response rates.

We conduct several checks on the representativeness and quality of our survey responses. In *SI Appendix*, section 1.C.1, we first ensure that our sample represents the population on observables, including age, gender, experience, earnings, and wealth. Second, following ref. 7, we use randomized participation incentives to show our findings are also robust to controlling for workers’ latent willingness to participate in the survey.

In *SI Appendix*, section 1.C.2, we cross-check that the survey responses align with variables that are also recorded in the administrative registers, including workers’ occupations and experiences. Furthermore, we show that repeat measures in the main survey and follow-up are strongly correlated.

## 2. Adoption of ChatGPT

### 2.1. Adoption across Occupations.

**Fig. 1.**
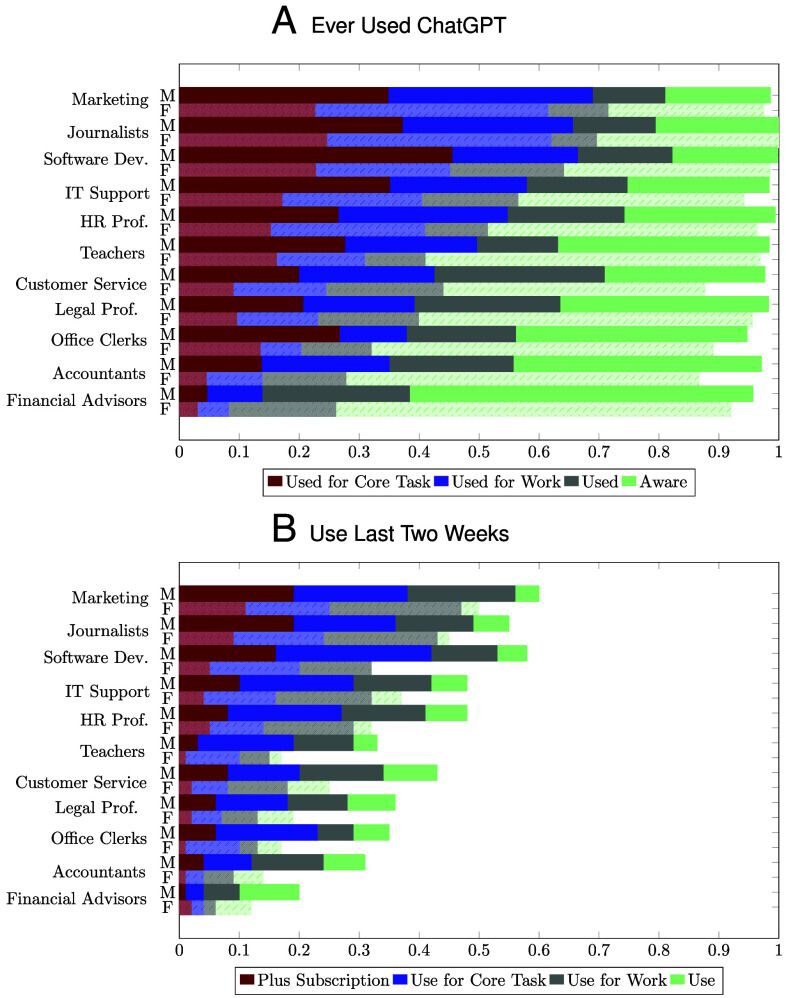
Adoption of ChatGPT by Occupations and Gender. This figure shows the adoption of ChatGPT in different occupations, split by male (M) and female (F) workers. Panel (*A*) plots the shares of workers who have used ChatGPT for a core job task, have used ChatGPT for work, have used ChatGPT, and are aware of ChatGPT. Panel (*B*) shows workers’ use of ChatGPT in the two past 2 wk, reporting whether they have an active Plus subscription to ChatGPT, use ChatGPT for a core job task, use ChatGPT for work, and use ChatGPT. “Core tasks” are “very important” or “extremely important” to the worker’s job. *Sample*: The figure is based on all completed survey responses.

Fig. 1 shows the adoption of ChatGPT across our 11 occupations, reporting whether workers have ever used it in Panel (*A*) and their use in the last 2 wk in Panel (*B*).

ChatGPT is widespread in the exposed occupations. More than half of workers have used it, 41% have used it for work, and 21% have used it for a core job task.^†^ Almost all workers are aware of ChatGPT.

Adoption rates differ across occupations, with marketing specialists and journalists—jobs where writing is essential—leading the curve with rates of adoption around 64%.^‡^ Occupations that handle sensitive information, such as financial advisors and accountants, have the lowest adoption rates, around 18%.

Not all workers who have ever used ChatGPT are still actively using it. In the last 2 wk, 34% have used it, 29% have used it for work, 16% have used it for a core task, and 7% have an active Plus subscription.

### 2.2. Adoption within Occupations.

Table 1 dives within the exposed occupations and asks what characterizes workers who have used ChatGPT for work.

**Table 1.** Who has used ChatGPT for work?

	Univariate					Multivariate	
	(1)	(2)	(3)	(4)	(5)	(6)	(7)
Age (10 y)	−0.070					−0.068	−0.048
	(0.003)					(0.004)	(0.006)
Experience (10 y)		−0.136				−0.063	−0.029
		(0.008)				(0.010)	(0.014)
log(Earnings)			0.022			0.082	0.047
			(0.007)			(0.009)	(0.012)
Net Wealth / Earnings				−0.021		−0.001	−0.002
				(0.001)		(0.002)	(0.002)
Female					−0.159	−0.138	−0.118
					(0.008)	(0.008)	(0.010)
Occupation FE’s	✓	✓	✓	✓	✓	✓	✓
Workplace FE’s							✓
Tasks FE’s							✓
Mean of Outcome	0.398	0.398	0.398	0.398	0.398	0.398	0.398
Observations	17,907	17,907	17,907	17,907	17,907	17,907	17,907

This table compares workers within occupations and asks what characterizes those who have used ChatGPT for work. Columns (1) to (5) represent univariate regressions, controlling for occupation fixed effects. Column (6) reports estimates from a multivariate regression that also controls for occupation fixed effects. Column (7) adds workplace and task importance FEs. SE in parentheses. All characteristics are based on register variables from 2022. Experience is the years of employment in the relevant occupation. Earnings are total labor income. Net Wealth is the sum of real assets, financial assets, and pension savings minus the sum of priority debt, other private debt, and public debt, winsorized at the 5th and 95th percentiles. *Sample*: The table is based on all completed survey responses that can be linked to the registry data.

Existing research highlights workers with less prior expertise have the most to gain from ChatGPT and other Generative AI (2, 3). Consistent with this view, we find younger and less experienced workers are more likely to use ChatGPT. In particular, every year of age and experience in the relevant occupation is associated with a 0.7 and 0.6 percentage point lower likelihood of having used ChatGPT for work.

However, despite the lower tenure, workers who use ChatGPT earned slightly more before its arrival, reflecting that more able workers are more likely to use ChatGPT.^§^ These adoption patterns suggest less able workers may need further assistance to reap the benefits of Generative AI.^¶^ Comparing the conditional correlations in Column (6) suggests that a 10 percentage point increase in ChatGPT usage can be achieved either by making a worker 15 y younger, 17 y less experienced, or by increasing his earnings by 122%.

The last row of Table 1 documents a staggering gender gap in the adoption of ChatGPT: Women are about 16 percentage points less likely to use ChatGPT than men in the same occupation. Fig. 1 shows the gender gap is pervasive across occupations, and *SI Appendix*, section 2.C.2 shows that it holds for a wide array of adoption measures.^#^

What explains the large gender differences in the use of ChatGPT? First, controlling for workers’ other characteristics in Column (6), the gender gap shrinks only slightly to 14 percentage points. Second, comparing workers within the same workplace and controlling for workers’ detailed task mixes in Column (7), the gender gap shrinks to 12 percentage points. Put differently, the lower use of ChatGPT among female workers is not primarily because they specialize in different job tasks or have other observable characteristics. In Section 4.4 below, we examine whether workers’ beliefs or adoption barriers drive the gender gap in the use of ChatGPT.

## 3. Beliefs about ChatGPT

Table 2 examines how workers anticipate ChatGPT will impact their job tasks.^‖^ Column (1) shows workers see a large productivity potential of ChatGPT in their occupations, estimating it can halve working times in about 37% of the job tasks for the typical worker.

**Table 2.** Worker beliefs about ChatGPT

			Expertise Complementarity				Cross-Task Substitution		
	Productivity (1)	Uncertain (2)	Negative (3)	Neutral (4)	Positive (5)	Individual Productivity (6)	Zero (7)	Inelastic (8)	Elastic (9)
Male	0.378	0.478	0.383	0.407	0.210	0.317	0.360	0.385	0.254
	(0.315)	(0.500)	(0.369)	(0.342)	(0.302)	(0.321)	(0.480)	(0.487)	(0.436)
Female	0.371	0.593	0.382	0.427	0.191	0.327	0.387	0.386	0.227
	(0.309)	(0.491)	(0.365)	(0.346)	(0.286)	(0.323)	(0.487)	(0.487)	(0.419)

This table shows male and female workers’ beliefs about ChatGPT (averages with SD in parentheses). Column (1) reports the share of job tasks where access to ChatGPT can halve working times for an average worker. Column (2) shows the share of tasks where workers are (very) uncertain about their productivity assessments [from Column (1)]. Columns (3) to (5) show the share of job tasks in which ChatGPT delivers respectively smaller, similar, and larger time savings for workers with greater task expertise. Column (6) shows the share of job tasks where access to ChatGPT can halve workers’ own working times. Column (7) shows the share of workers who will not complete more of a task if ChatGPT can save time in it. Column (8) is the share of workers who will complete more of a task but will not dedicate a larger share of their work time to the task. Column (9) is the share of workers who will dedicate a larger share of their time to a task if ChatGPT can save time completing it. *Sample*: Columns (1) to (5) are based on all completed survey responses. Columns (6) to (9) focus on the control group, as these survey questions come after the treatment page.

Table 2, Columns (3) to (5) report workers’ beliefs about whether ChatGPT provides smaller, similar, or larger time savings for workers with more expertise in the task. Workers are about twice as likely (38% vs. 20%) to state the time savings from ChatGPT are smaller rather than larger for workers with greater task expertise. These patterns align with existing research on the productivity effects of ChatGPT (2, 10) and suggest workers understand the technology substitutes for human expertise.

Table 2, Column (6) shows how workers perceive their own time savings from ChatGPT. Workers are slightly more skeptical about their own productivity gain from ChatGPT [compared with the typical worker in Column (1)], estimating it can halve their working times in about 32% of their job tasks. *SI Appendix*, section 3 shows workers give their expertise levels (especially among men) and worries about correctness (especially among women) as the main reasons they gain less from ChatGPT than the average worker.

Table 2, Columns (7) to (9) show how workers expect ChatGPT will impact their task outputs and time allocations. Strikingly, about 37% of workers report they will not perform more of a task if ChatGPT can save time completing it. By contrast, about 24% of workers report they will devote a larger share of their working time to tasks ChatGPT can save time completing.^**^ The limited cross-task substitution suggests that in the short run, before industries have reorganized work around the new technology, ChatGPT may cause limited reallocation between job tasks.^††^

Finally, Table 2 reveals that beliefs about ChatGPT vary vastly within occupations: the SD of workers’ estimated productivity shares is around 31 percentage points [Column (1)]. Furthermore, most workers are (very) uncertain about the time savings from ChatGPT [Column (2)], with women being especially uncertain about their assessments.

## 4. Beliefs vs. Adoption

How do workers’ perceived benefits from ChatGPT relate to their usage of the technology? First, workers who have already used ChatGPT may be more knowledgeable about its capabilities. Conversely, workers who are more optimistic about the capabilities of ChatGPT may be more inclined to use it. *SI Appendix*, section 4 examines the links between workers’ use of ChatGPT and their beliefs about it. We summarize the key takeaways below.

### 4.1. Prior Use, Beliefs, and Intended Use.

*SI Appendix*, section 4.A shows that workers see a substantial productivity potential in ChatGPT regardless of their actual experiences with the tool, with estimated productivity shares only slightly higher among workers who have actually used it (32.6% vs. 30.6%).

Workers who have not used ChatGPT at this point also do not intend to use it going forward. For example, even in the tasks where these “never users” say ChatGPT could halve their working times, only 3.3% intend to use it within the next 2 wk.

More generally, workers’ estimated time savings from ChatGPT are only a weak predictor of their use of the tool. Even among workers who have used ChatGPT before and estimate it can half working times in a job task, only 36.3% intend to use it.

### 4.2. Adoption Frictions.

*SI Appendix*, section 4.B examines why workers do not intend to use ChatGPT despite stating it could half their working times in tasks they perform. The most important reasons relate to firm policies: 42% of the workers report they need training to use ChatGPT, and 36% report employers actively restrict their usage. “Existential fears” of becoming redundant in the job or dependent on technology are the least important adoption frictions, with less than 9% of workers reporting these fears as reasons for not using ChatGPT despite its time savings.

Frictions to adoption help explain the systematic differences between occupations in the use of ChatGPT (Fig. 1). For example, while 82% of financial advisors who report large time savings from ChatGPT face an adoption friction, only 35% of software developers report the same. The relevant frictions also differ by occupation. Employer restrictions are more likely to bind in occupations that handle sensitive information, such as financial advisors and legal professionals. Less IT-prone occupations, such as teachers, report they need training to use ChatGPT, whereas this is less of a concern for software developers. Customer service representatives avoid ChatGPT due to fears of being replaced or becoming dependent on technology. Finally, in occupations where writing is a core competency, such as journalism and teaching, workers resist ChatGPT because it diminishes their enjoyment of their jobs.

The reported need for training may seem surprising since ChatGPT does not require special technical training. Part of the stated need for training could reflect a reluctance to try it out, as the friction diminishes by 28% when focusing on workers who have actually tried the tool. Still, even among workers who have used the tool and state it could halve working times, 13% report they need training before using it on the job.

Finally, it is worth noting that the stated adoption frictions relate to workers’ intended use, and that individuals’ intentions often fail to manifest in behaviors (13). Indeed, while the correlation between workers’ estimated time savings and intended use is already weak at 27%, the correlation with their actual prior use is even lower at 19%.

### 4.3. Information Treatment.

What is the causal role of worker beliefs in the adoption of ChatGPT? To evaluate this question, *SI Appendix*, section 4.C exposes a random set of participants to expert assessments of the time savings from ChatGPT.

The information treatment is successful in shifting workers’ beliefs, with effects that persist in the follow-up survey 2 wk later. Yet, despite shifting beliefs, the information treatment has muted effects on workers’ intended use of ChatGPT and no effects on their actual use of the tool in the 2 wk after.

In our context, the muted effects on adoption are consistent with the frictions documented in Section 4.2 as workers point to employer restrictions and the need for training as their reasons for not reacting to the shifted beliefs. More generally, information provision experiments often fail to affect behavior (14).

### 4.4. Gender Gaps.

**Fig. 2.**
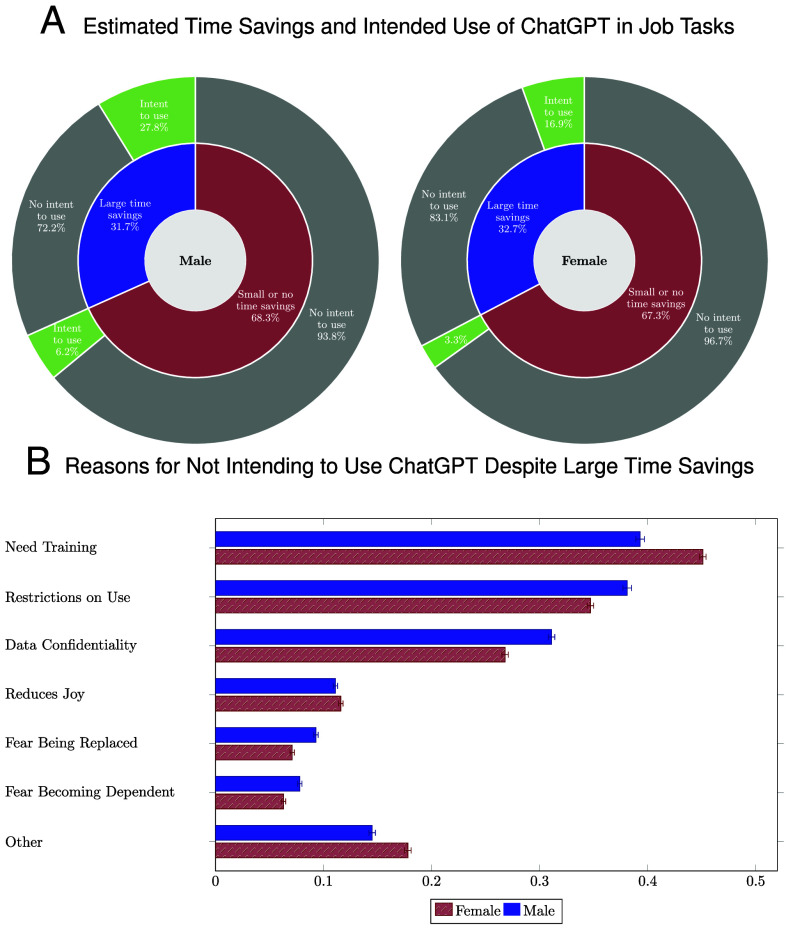
Beliefs, Intended Use, and Frictions by Gender. This figure shows the relationship between workers’ estimated time savings from ChatGPT, intended use of the tool, and adoption frictions in their job tasks. The figures are split by male and female workers. The figure assigns each occupation with a uniform weight for each gender, ensuring that any gender differences do not reflect differences in occupational compositions. Panel (*A*) shows the relationship between workers’ estimated time savings from the ChatGPT in their job tasks (inner ring) and their intended use of ChatGPT (in the coming 2 wk) in the job tasks (outer ring). Panel (*B*) shows workers’ reported reasons for not intending to use ChatGPT despite stating it could generate large time savings. The figure focuses on tasks workers expect to perform in the coming 2 wk. The excluded job tasks (that workers do not expect to perform) constitute 19% (23%) of the tasks with large reported time savings by male (female) workers. As workers may report multiple reasons for their friction, the reason bars may sum to more than 100%. Whiskers represent 95% confidence bands. *Sample*: The figure is based on all completed survey responses of the control group.

Why have so few women adopted ChatGPT? In Fig. 2, we examine the roles of worker beliefs and adoption frictions in driving the gender gap in the use of ChatGPT.

The gender gap in adoption does not reflect differences in average beliefs, as women are about as optimistic as men about the time savings from ChatGPT [inner ring of Panel (*A*)].^‡‡^ Furthermore, women’s use of ChatGPT is slightly better calibrated to the time savings than men’s [outer ring of Panel (*A*)].^§§^

Women are more likely to face adoption frictions, however, especially related to training. Panel (*B*) shows workers’ reported reasons for not intending to use ChatGPT despite stating it could halve working times in job tasks they perform. These job tasks represent, respectively, 60% and 53% of the tasks with large reported time savings by female and male workers.^¶¶^ Among the affected workers, 45% of women report they need training to use ChatGPT. By contrast, men’s use is more often limited by employer restrictions and data confidentiality. (*SI Appendix*, section 3 shows women are also more likely to state they “do not know how” to use ChatGPT as a reason their benefits from it are lower.)^,^ [Carvajal et al. (15) identify a comparable gender gap in a survey experiment among 514 university students in Norway. In particular, they document female students use ChatGPT much less, are less proficient at writing ChatGPT prompts, and are more sensitive to bans on using ChatGPT.]

Finally, the gender gap in adoption does not reflect women’s beliefs are less responsive to information about the technology. On the contrary, *SI Appendix*, section 4.C.5 shows that women respond more to the information treatment but face barriers that prevent their adoption.

## 5. Conclusion

The arrival of ChatGPT is a landmark event in technology history. A year after its launch, ChatGPT is widespread in the exposed occupations, yielding substantial time savings for users. This paper documents how obstacles to its adoption have reinforced existing inequalities, with women and lower-earning workers less likely to use ChatGPT. Our findings are based on a large-scale and representative survey linked to comprehensive register data in Denmark.

Thus far, the rapid take-up has been driven by the individual decisions of workers to start using it, with many employers playing a passive or regressive role. Looking ahead, firms could play a critical role in facilitating the further adoption of Generative AI such as ChatGPT. Indeed, many workers who currently do not use ChatGPT report employers are restricting their use or that they need training to use it. Hence, by providing guidelines for productive use or facilitating employee training, employers could help more workers unlock the productivity potential of Generative AI.

A proactive approach by firms or governments to aid the further adoption of Generative AI could also help alleviate three concerning patterns in the current adoption of ChatGPT. First, despite the potential of Generative AI to alleviate existing inequalities, workers who currently use ChatGPT earned slightly more before its arrival. Hence, workers with less expertise may need further assistance to reap the benefits of Generative AI.

Second, our analysis revealed a staggering gender gap in adoption, with women much less likely to use ChatGPT. A planned effort to train workers could help resolve this gender gap, as many women report they need training to use ChatGPT.

Finally, many workers report they will not expand their output in tasks where ChatGPT boosts their productivity. However, as firms reorganize their workflows around Generative AI such as ChatGPT, the productivity gains may also deliver greater expansion in output, ultimately contributing to economic growth.

## Supplementary Material

Appendix 01 (PDF)

## Data Availability

Programs data will be deposited in Open Science Framework. Data cannot be shared. (Our study uses proprietary microdata from the research servers of Statistics Denmark. Interested researchers can apply for data access through the Research Service department at Statistics Denmark: https://www.dst.dk/en/TilSalg/Forskningsservice/Dataadgang.) (16)

## Footnotes

^*^Because the O*NET database only covers existing tasks, our survey will tend to focus on the automation potential of ChatGPT. Autor et al. (5) and Acemoglu and Restrepo (6) show that new technologies often also create new tasks (e.g., fact-checking of GPT-generated output), which tend to reinstate workers in the production process.

^†^*SI Appendix*, section 2.B.1 shows that most workers who use ChatGPT for work also use it for leisure and that workers are equally likely to use ChatGPT “core” vs. peripheral” job tasks.

^‡^*SI Appendix*, section 2.B.2 shows our rates of adoption align with smaller-scale national surveys of ChatGPT, and that the adoption rates for ChatGPT are high relative to existing AI technologies. The faster adoption of ChatGPT likely reflects that it has been driven by the take-up of individual workers independent of firm-wide initiatives.

^§^*SI Appendix*, section 2.C.1 shows educational achievements do not seem to drive the ability bias as workers’ years of schooling and high school GPAs are uncorrelated with their use of ChatGPT for work.

^¶^Otis et al. (8) show high-performing entrepreneurs in Kenya accrue larger gains from access to a GPT-powered AI assistant, consistent with our finding that higher-achieving workers may be better positioned to use ChatGPT.

^#^*SI Appendix*, section 2.B.2 shows that it is difficult to find another technology (past or present) with a similar gender gap as ChatGPT, especially within detailed occupations.

^‖^Acemoglu (9) show how task-level adoption rates (Fig. 1), time savings (Table 2, Columns 1 to 6), and cross-task substitutions (Table 2, Columns 7 to 9) determine the macroeconomic impacts of AI.

^**^*SI Appendix*, section 3 shows the cross-task substitution is particularly limited among teachers, among whom 61% state a cross-task substitution of zero, whose job tasks arguably are more fixed by the school system.

^††^Eisfeldt et al. (11) show firms exposed to ChatGPT saw a rise in their stock prices following its release, reflecting anticipations that the technology will help reduce their labor costs. Agrawal et al. (12) provide a model of organization-wide adoption of AI across job tasks. The reorganization of work to leverage tools like ChatGPT may also create new tasks for workers, further boosting labor demand (5, 6).

^‡‡^Women are more uncertain about these assessments, however, and controlling for uncertainty of beliefs shrinks the residual gender gap in adoption from 11.8 to 10.4 pp; see *SI Appendix*, section 2.C.3.

^§§^Women’s intended adoption is 3.4 times higher in tasks with large compared to small time savings from ChatGPT. This ratio is 3.0 for men.

^¶¶^The difference to the (“Large time savings” & “No intent to use”) segments of Fig. 2*A* reflects job tasks that workers do not expect to perform within the next 2 wk (23% for women, 19% for men).
