# Supplementary material for: The unequal adoption of ChatGPT exacerbates existing inequalities among workers
Source: Proc Natl Acad Sci U S A. 2024 Dec 30;122(1):e2414972121. doi: 10.1073/pnas.2414972121 (PMC11725873; doi:10.1073/pnas.2414972121)
Supplement: Supplementary file 1 — Appendix 01 (PDF) [file pnas.2414972121.sapp.pdf]

Supporting Information for

The unequal adoption of ChatGPT exacerbates existing inequalities among workers

Anders Humlum, Emilie Vestergaard

Anders Humlum.
E-mail: anders.humlum@chicagobooth.edu

This PDF file includes:

Figs. S1 to S5
Tables S1 to S25
SI References

1. Materials and Methods

1.A. Expert Assessments. The starting point of our study is an expert assessment of the time savings from ChatGPT in detailed job tasks. We use the expert assessments (*i*) to identify occupations that are exposed to ChatGPT and (*ii*) as the information treatment in our experiment.

1.A.1. Productivity Measure. Our expert assessments are based on the "Direct Exposure (E1)" measure of (1), which asks whether access to ChatGPT can halve the time an average worker takes to complete a task at equal quality. The measure is based on the capabilities of GPT-3.5, which powered the free version of ChatGPT at the time of our study.* The box below provides the rubric of the measure, which we call "productivity" in this paper.

Productivity Rubric (1)

Think of a **[journalist] with an average level of experience and expertise** trying to complete a given task. The worker has access to ChatGPT, the internet, a computer with existing software, and other tools typically used to complete the task.

Specify the following tasks according to the rubric below. Equivalent quality means someone reviewing the work would not be able to tell whether the worker completed it with or without assistance from ChatGPT.

Large time savings from ChatGPT
Specify the task's time savings as "Large" if **access to ChatGPT** ***can halve the time*** it takes for an average [journalist] to complete the task with equivalent quality.

Small or no time savings from ChatGPT
Specify the task's time savings as "Small or no" if **access to ChatGPT** ***cannot halve the time*** it takes for an average [journalist] to complete the task with equivalent quality.

[***Write commentaries, columns, or scripts***]

This figure provides the rubric for our *productivity* measure.

1.A.2. GPT Ratings. (1) use a combination of human assessments and GPT prompts to classify the productivity of ChatGPT in the Detailed Work Activities (DWAs) in the O*NET database. We start by replicating the GPT ratings of (1), applying minor adjustments to classify the most detailed Job Duties in the O*NET register.†,‡ We validate that the GPT ratings match our independent assessments of a random selection of 100 tasks.

1.A.3. Selecting Occupations. We use the GPT ratings to identify the occupations to include in our study. In particular, we include all occupations that (*i*) have at least one job task that is exposed to ChatGPT, (*ii*) are captured by a well-defined set of occupational (ISCO) codes, and (*iii*) contain enough workers for statistical analysis. We complement the occupational codes with industry codes (DB, a disaggregation of NACE) and educational codes (HFAUDD) to help identify the target occupations in the registers. We measure the 11 survey occupations as follows:

1. Accountants and Auditors: ISCO 2411, 3313.
2. Customer Service Representatives: ISCO 4222, 4225, 4229.
3. Financial Advisors: ISCO 2412 and DB 641900.
4. Human Resource Professionals: ISCO 2423, 4416.
5. IT-support workers: ISCO 351.
6. Journalists: ISCO 264, DB 581300, 581410, 581420, 601000, 602000.
7. Lawyers and Paralegals: ISCO 2611, 2619, 3411.
8. Marketing Professionals: ISCO 2431, 2433, 2434.
9. Secretaries and Office Clerks: ISCO 334, 411, 412.
10. Software Developers: ISCO 251, DB 620000-620900.
11. Teachers
    (a) Primary school: ISCO 2341, DB 852010, HFAUDD 5440, 5441.
    (b) High school: ISCO 233, DB 853120.

1.A.4. Selecting Job Tasks. We include six job tasks for each occupation in our survey. We select the job tasks to represent ChatGPT's average capabilities in the entire set of job tasks in the occupations (the O*NET database typically contains 20-50 job tasks per occupation). In addition to matching the average productivity scores (the E1 score of (1)), we also ensure the job tasks are representative with respect to expertise-complementarity (expert assessment of whether ChatGPT delivers smaller/similar/larger time savings for workers with greater expertise) and the forward-looking productivity measures E2 ("Exposure by LLM-powered applications") and E3 ("Exposure given image capabilities") of (1).

* (1) also propose the forward-looking measures "Exposure by LLM-powered applications (E2)" and "Exposure given image capabilities (E3)." We use the E1 scores as these capture the technology available to workers at the time of our study.

† We thank Pamela Mishkin and Daniel Rock for sharing their GPT prompt and exposure scores.

‡ (1) classify the DWAs in O*NET, as these are comparable across occupations. We use the Job Duties because they are more relevant to specific occupations. In total, we have around 30,000 Job Duties that aggregate up to approximately 1,600 DWAs.

Define scores X_t of tasks t to target

$$X = \{\text{E1}(0/1), \text{E2}(0/1), \text{E3}(0/1), \text{Complementarity}(-1/0/1)\}. \quad [1]$$

Our goal is to find a combination of six job tasks t whose average scores X match those in the entire set of job tasks $\mathcal{T}_o$ of occupation o.

First, to address the fact that the scores above are not measured on the same scale, we calculate distances in standard deviations of each score (calculated among all tasks of occupation o). Second, to allow some scores to receive higher priority, we assign each score a weight $\omega = (\omega_1, ..., \omega_S)$, where $S = \#M$.

Define all combinations of six job tasks drawn from $\mathcal{T}_o$ without replacement by $\mathcal{C}(\mathcal{T}_o)$. Our objective function reads

$$\min_{\mathcal{T}_c \in \mathcal{C}(\mathcal{T}_o)} \sum_{s=1}^{S} \omega_s \frac{(m_c(X_s) - m_{Po}(X_s))^2}{v_{Po}(X_s)}, \quad [2]$$

where $m_c(X)$ is the mean score of task combination c, and $m_{Po}(X)$ and $v_{Po}(X)$ are the population mean and variances of scores in occupation o.

We solve equation Eq. (2) by evaluating the objective function at all combinations of job tasks. For each occupation, we successfully find a task selection that exactly matches the productivity and complementarity scores. In the case of multiple minima of Equation Eq. (2), we prioritize job tasks with higher O*NET importance scores for the occupation.

Section 6 lists the resulting job tasks we include for each occupation in the survey.

1.A.5. Human Ratings. We independently rate each of the six job tasks in the 11 occupations. The GPT and human ratings agree on all of the 66 job tasks. We furthermore validate the assessments with industry specialists and technology experts in Denmark.

1.A.6. Explanations and Sheets. Our information treatment includes a short (1-2 sentence) explanation for each expert assessment. We use GPT to generate draft explanations for its assessed productivity ratings, which we manually review for validity and clarity. Section 9 provides our final set of expert assessments and explanations. Furthermore, we allow participants to sign up for information sheets on how to use ChatGPT in their job tasks. Section 10 provides details on the sheets.

1.B. Survey Outline.

1.B.1. Main Survey. Our main survey is organized into five blocks summarized below.

Block 1: Adoption. After selecting their occupation, workers are asked about their experiences with ChatGPT. Workers report the importance of the surveyed tasks in their jobs and their own expertise in each job task.

Block 2: Prior Beliefs. Workers assess the time savings from ChatGPT for an average worker in their occupation, thus completing the productivity rubric of Section 1.A.1.[§] Workers also assess whether the time savings are smaller, similar, or larger for workers with greater expertise in the task.

Block 3: Treatment. The treatment group is exposed to the expert assessments from Section 1.A, seeing a comparison of their assessments and the expert assessments, together with a brief explanation of each expert assessment. The control group sees a summary of their assessments. Figure S5 displays the treatment and control pages.

Block 4: Intended Adoption and Posterior Beliefs. Workers report their intended use of ChatGPT in the coming two weeks. They also assess their own time savings from ChatGPT, thus completing the productivity rubric for themselves (instead of the average worker), which we call "individual productivity". Finally, workers assess how time savings from ChatGPT will affect their task outputs and time allocations.

Block 5: Frictions. If applicable, workers are asked why their assessed time savings from ChatGPT differ for an average worker versus themselves. If applicable, workers are asked why they do not intend to use ChatGPT despite stating it could save them time. Workers may also sign up for an information sheet with use cases of ChatGPT in their job tasks.

1.B.2. Follow-Up Survey. We distribute a follow-up survey two weeks after workers' responses to the main survey. The follow-up survey follows the structure of the main survey with two exceptions: in Block 1, we ask about adoption and task importance in the past two weeks. We ask this question to be consistent with the time window of intended adoption in Block 4 of the main survey. Second, we exclude Block 3 (Treatment) from the follow-up survey.

[§] Incentivized belief elicitation is not possible because Statistics Denmark does not allow payments that depend on respondents' answers.

1.C. Survey Sample. Table S1 shows our analysis sample. We invited about 100,000 individuals to participate in our main survey in November 2023. The survey achieved a gross response rate of 29.2%, among which 86.4% (=18,109/25,121) were still employed in an exposed occupation. The remaining 13.6% had left their jobs between June 2023 (the latest month of register data) and November 2023 (the launch of the survey) or whose occupational codes were outdated in the registers. The main survey had a completion rate of 72.1% (=18,109/25,121), among which 98.9% (=17,909/18,109) could be linked to our register variables in Table 1 in the main text.

Of the workers who completed the main survey, 41.0% responded to our follow-up survey, with a completion rate of 81.2% (=6,029/7,425). Attrition rates in our survey are balanced across our treatment arms and similar to previous surveys in the Danish setting (2). Although we focus our main analysis on the completed responses, Section 1.C.1 shows all our findings are robust to adding in the partial responses.

Table S1. Analysis Sample Reduction

		Individuals (1)	Percent of invitees (2)
A.	Main Survey		
1.	Invitees	99619	100
2.	Respondents	29067	29.2
3.	In target occupation	25121	25.2
4.	Complete responses	18109	18.2
5.	Linked to registers	17907	18
B.	Follow-Up Survey		
1.	Invitees	18109	100
2.	Respondents	7700	42.5
3.	Complete responses	6029	33.3
4.	In productivity or control groups	4052	22.4

This table shows how our analysis sample shrinks with sample restrictions. Panel A focuses on the main survey. Row 1 reports the total number of invitations. Row 2 reports the individuals who responded to our surveys. Row 3 shows the respondents who were still employed in one of our 11 target occupations. Row 4 reports the respondents who fully completed the survey. Row 5 reports the complete responses we can link to the register variables in Table 1 in the main text. Panel B focuses on the follow-up survey. Row 1 shows we invited all individuals who had fully completed the main survey (Row A.4) to participate in the follow-up. Row 2 shows the individuals who responded to the follow-up survey. Row 3 reports the respondents who fully completed the follow-up survey. Row 4 shows the complete responses of the "productivity treatment" or control groups studied in Table S20.

1.C.1. Representativeness. In this section, we conduct checks to ensure our survey data paint a representative picture.

First, Table S3 ensures our sample represents the population on observables, including age, gender, experience, earnings, and wealth, and Table S2 (Column (2)) shows our findings are robust to controlling for selection into the survey based on these observables.

Second, following (3), we use randomized participation incentives to examine selection into our survey based on worker unobservables. Table S4 shows workers who randomly receive a higher participation prize are more likely to participate in the survey but do not systematically differ in their responses. Table S2 (Column (3)) uses this variation to show our findings are robust to controlling for workers' latent willingness to participate in the survey.

Finally, we compare the responses of workers who fully complete versus drop out of our main survey and follow-up. Table S5 shows that these workers have similar characteristics, adoption behaviors, and beliefs about ChatGPT.

Table S2. Robustness to Non-Response Bias

	Raw<br>(1)	Observables<br>(2)	Unobservables<br>(3)
Panel A: Adoption			
Used	0.553	0.577	0.539
	(0.497)	(0.494)	(0.497)
Used for Work	0.399	0.414	0.405
	(0.490)	(0.493)	(0.490)
Used for Core Task	0.215	0.217	0.209
	(0.410)	(0.412)	(0.411)
Panel B: Beliefs			
Productivity	0.364	0.380	0.425
	(0.310)	(0.311)	(0.310)
Negative Complementarity	0.381	0.376	0.349
	(0.368)	(0.365)	(0.368)
Neutral Complementarity	0.424	0.424	0.426
	(0.348)	(0.345)	(0.348)
Positive Complementarity	0.195	0.200	0.224
	(0.291)	(0.293)	(0.291)
Individual Productivity	0.309	0.326	0.308
	(0.318)	(0.322)	(0.318)
Zero Substitution	0.397	0.376	0.315
	(0.489)	(0.484)	(0.489)
Inelastic Substitution	0.381	0.396	0.475
	(0.486)	(0.489)	(0.485)
Elastic Substitution	0.222	0.228	0.210
	(0.416)	(0.420)	(0.416)

This table shows how accounting for non-response bias affects summary statistics of workers' adoption of and beliefs about ChatGPT. Column (1) shows the raw means of the survey responses. Column (2) reweighs the survey responses according to workers' inverse probability of survey participation based on a logit regression on the observables in Table 1 in the main text (including occupation). Column (3) reweighs the survey responses according to workers' latent resistance to survey participation; see Section 5.A for details on the method. Standard deviations in parentheses. *Sample*: The table is based on all completed survey responses.

Table S3. Balance Table for Survey Respondents

	Population<br>(1)	Sampled<br>(2)	Responded<br>(3)
Age	42.43	42.42	45.40
	(11.57)	(11.57)	(11.50)
Female	0.52	0.52	0.49
	(0.50)	(0.50)	(0.50)
log(Earnings)	13.07	13.07	13.11
	(0.58)	(0.59)	(0.53)
Experience	6.05	6.05	7.12
	(4.58)	(4.57)	(4.67)
Wealth / Earnings	4.09	4.87	4.10
	(157.40)	(262.31)	(39.57)
Observations	283,399	99,817	18,088

This table compares the mean characteristics of workers (variables from Table 1 in the main text) among our population (Column 1), our sampled survey invitees (Column 2), and the survey respondents (with complete responses, Column 3), with standard deviations in parentheses. *Sample*: The table is based on all individuals in our survey population.

Table S4. Balance Table for Participation Prize Categories

	Levels	Differences to 1000 DKK			
	1000 DKK	2500 DKK	5000 DKK	10000 DKK	p-value
	(1)	(2)	(3)	(4)	(5)
Panel A: Characteristics					
Age	45.40	-0.46	-0.43	-0.50	0.14
		(0.24)	(0.84)	(0.85)	
log(Earnings)	13.11	-0.03	-0.00	-0.01	0.04
		(0.01)	(0.05)	(0.05)	
Experience	7.12	-0.01	-0.01	-0.05	0.95
		(0.09)	(0.35)	(0.35)	
Net Wealth/Earnings	4.10	-0.05	0.87	0.38	0.55
		(0.27)	(0.42)	(0.42)	
Female	0.49	0.00	-0.01	-0.00	0.44
		(0.01)	(0.03)	(0.03)	
Panel B: Adoption					
Used	0.55	-0.02	-0.01	-0.01	0.42
		(0.01)	(0.03)	(0.03)	
Used for Work	0.40	-0.01	-0.00	-0.00	0.63
		(0.01)	(0.03)	(0.03)	
Used for Core Task	0.21	-0.01	0.00	-0.00	0.59
		(0.01)	(0.02)	(0.03)	
Panel C: Beliefs					
Productivity	0.36	0.00	0.01	0.01	0.10
		(0.01)	(0.02)	(0.02)	
Negative Complementarity	0.38	-0.01	-0.01	-0.01	0.44
		(0.01)	(0.03)	(0.02)	
Neutral Complementarity	0.42	0.01	0.01	0.00	0.33
		(0.01)	(0.02)	(0.02)	
Positive Complementarity	0.19	-0.00	0.00	0.01	0.70
		(0.01)	(0.02)	(0.02)	
Individual Productivity	0.31	-0.02	0.00	-0.00	0.36
		(0.01)	(0.03)	(0.03)	
Zero Substitution	0.40	0.01	-0.03	-0.01	0.16
		(0.02)	(0.07)	(0.07)	
Inelastic Substitution	0.38	-0.02	0.02	0.02	0.19
		(0.02)	(0.07)	(0.07)	
Elastic Substitution	0.22	0.01	0.01	-0.00	0.80
		(0.02)	(0.05)	(0.06)	
Response Rate	0.16	0.02	0.02	0.04	0.00
		(0.00)	(0.00)	(0.00)	
Observations	4,021	4,518	4,547	5,002	

This table shows individuals assigned to the different participation prize categories (1,000 DKK, 2,500 DKK, 5,000 DKK, and 10,000 DKK) have similar characteristics (Panel A), adoption behaviors (Panel B), and beliefs (Panel C) but differ in their rates of completed responses (last row). Column (5) reports p-values of a joint test that the mean outcomes are equal across the four prize categories. Table S2 uses the differences in take-up to account for non-response bias in the survey responses; see Section 5.A for details. *Sample*: The table is based on all completed survey responses.

Table S5. Balance Table for Complete vs. Partial Responses

	Main Survey		Follow Up Survey	
	Completed	Drop Out	Completed	Drop Out
	(1)	(2)	(3)	(4)
Panel A: Characteristics				
Age	45.40	45.01	46.80	45.37
	(11.50)	(11.52)	(11.38)	(11.30)
log(Earnings)	13.11	13.10	13.13	13.15
	(0.53)	(0.53)	(0.50)	(0.47)
Experience	7.12	6.88	7.62	7.29
	(4.67)	(4.63)	(4.65)	(4.58)
Net Wealth/Earnings	4.10	3.75	5.20	3.23
	(39.57)	(16.43)	(64.01)	(11.94)
Female	0.49	0.60	0.47	0.52
	(0.50)	(0.49)	(0.50)	(0.50)
Panel B: Adoption				
Used	0.55	0.51	0.54	0.59
	(0.50)	(0.50)	(0.50)	(0.49)
Used for Work	0.40	0.38	0.38	0.44
	(0.49)	(0.48)	(0.49)	(0.50)
Used for Core Task	0.21	0.17	0.20	0.24
	(0.41)	(0.38)	(0.40)	(0.43)
Panel C: Beliefs				
Productivity	0.36	0.37	0.35	0.38
	(0.31)	(0.32)	(0.31)	(0.31)
Negative Complementarity	0.38	0.33	0.39	0.38
	(0.37)	(0.35)	(0.37)	(0.36)
Neutral Complementarity	0.42	0.44	0.42	0.42
	(0.35)	(0.36)	(0.35)	(0.34)
Positive Complementarity	0.19	0.22	0.19	0.20
	(0.29)	(0.31)	(0.29)	(0.29)
Observations	18,088	7,003	6,515	1,775

This table compares the mean characteristics adoption behaviors, and beliefs (in the main survey) of workers who fully complete the main survey (Column 1), partially complete the main survey (Column 2), fully complete the follow-up survey (Column 3), and partially complete the follow-up survey (Column 2). Standard deviations in parentheses. *Sample*: The table is based on all individuals with partial responses to our main survey.

1.C.2. Response Quality. In this section, we examine the quality of our survey responses.

As an external validation, we cross-check variables that are also recorded in the administrative registers. First, our survey and administrative registers agree on the occupation of 87% of our respondents.[¶] Second, validating the quality of respondents' subjective assessments, Figure S1 shows workers' self-reported task expertise is strongly correlated with their experience in the relevant occupation recorded in the registers.

As an internal validation, we examine the correlation between repeat measurements in the main survey and the follow-up survey. In particular, Tables S8 and S9 show that workers' reported task importance and task expertise scores are strongly correlated between the main survey and follow-up. Further validating workers' assessments, Table S7 shows that workers' reported expertise in a task is highly related to how important the task is in their jobs.

Finally, we confirm workers' beliefs about ChatGPT correlate with the expert assessments. Workers and experts agree on the exposure rankings of 78% of the job tasks. For example, according to workers, the least exposed job task is "Establish and enforce rules for behavior and procedures for maintaining order among students" of teachers, and the most exposed task is "Analyze financial information obtained from clients to determine strategies for meeting clients' financial objectives" of financial advisors.

Table S6. Correlation between Occupation in Survey vs. Register, $P(\text{Survey}|\text{Register})$

		In Survey											
		Journalists	Software Developers	Paralegals	Accountants and Auditors	Customer Service Rep.	Marketing Professionals	Financial Advisors	HR Professionals	Office Clerks	Teachers	IT Support	Observations
Register	Journalists	0.97	0.00	0.00	0.00	0.00	0.01	0.00	0.00	0.01	0.00	0.00	555.00
	Software Developers	0.00	0.87	0.00	0.00	0.01	0.02	0.00	0.00	0.01	0.00	0.08	3,185.00
	Paralegals	0.01	0.03	0.79	0.02	0.01	0.00	0.01	0.02	0.08	0.01	0.01	2,518.00
	Accountants and Auditors	0.00	0.02	0.01	0.85	0.01	0.01	0.02	0.02	0.05	0.00	0.01	2,710.00
	Customer Service Rep.	0.01	0.03	0.01	0.01	0.79	0.04	0.01	0.01	0.07	0.01	0.01	869.00
	Marketing Professionals	0.00	0.05	0.00	0.00	0.09	0.74	0.01	0.01	0.06	0.00	0.03	2,125.00
	Financial Advisors	0.00	0.00	0.00	0.00	0.01	0.00	0.95	0.00	0.02	0.00	0.00	1,918.00
	HR Professionals	0.01	0.03	0.06	0.01	0.00	0.01	0.02	0.68	0.14	0.01	0.02	1,434.00
	Office Clerks	0.00	0.01	0.00	0.00	0.00	0.01	0.00	0.00	0.96	0.00	0.01	3,395.00
	Teachers	0.00	0.00	0.00	0.00	0.00	0.00	0.00	0.00	0.00	1.00	0.00	4,135.00
	IT Support	0.00	0.15	0.00	0.00	0.02	0.02	0.00	0.01	0.03	0.00	0.76	2,277.00

This table shows the correlation between the occupational codes reported in the survey and those registered in the administrative data of Statistics Denmark. The cells show the probability of reporting the column occupation in the survey, conditional on having the row occupation registered with Statistics Denmark. The average agreement rate (diagonal element) is 87%. *Sample*: The table is based on all completed survey responses.

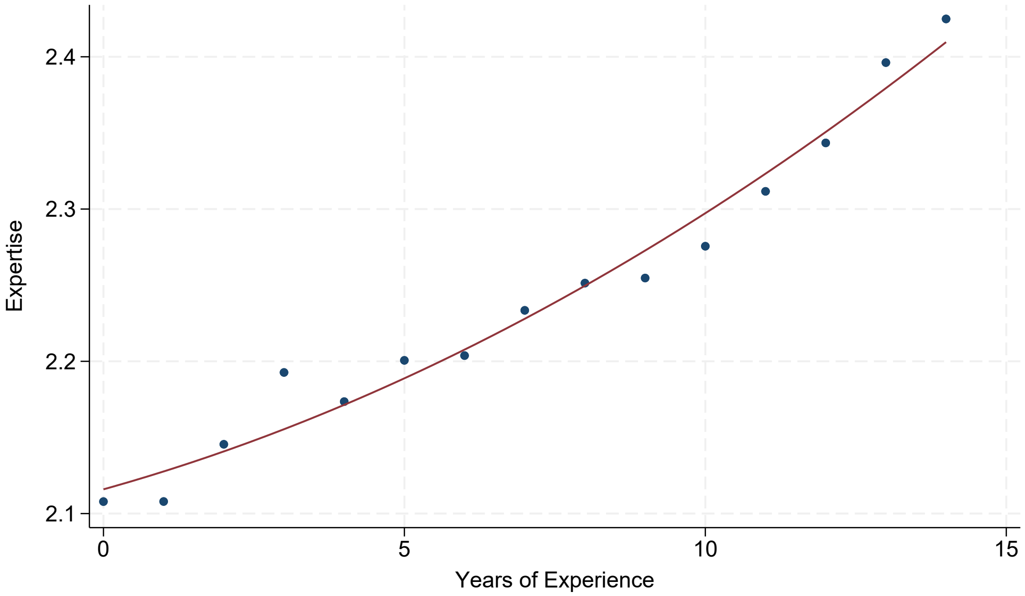

Fig. S1. Self-Reported Task Expertise vs. Registered Experience in Occupation. This figure shows the correlation between workers' self-reported expertise and their years of experience in the relevant occupation. Expertise is reported on a three-point scale: 1 (Low), 2 (Average), and 3 (High). *Sample*: The table is based on all completed survey responses that can be linked to registry data on experience.

[¶] The disagreements likely reflect measurement error in the registers because firms generally do not update occupational switches of existing employees (4). Furthermore, some workers may have switched jobs between June 2023 (our latest month of register data) and November 2023 (the launch of our survey). Table S6 shows the disagreements occur in cells that reflect likely switches, such as (IT Support, Software Developer). By contrast, the survey and register data agree on the occupation of 100% of our school teachers.

Table S7. Self-Reported Expertise vs. Task Importance

		Expertise			
		Low	Average	High	Observations
Importance	Not Important	0.68	0.22	0.09	15,816
	Somewhat Important	0.35	0.48	0.17	13,121
	Important	0.13	0.54	0.32	24,203
	Very Important	0.05	0.39	0.55	29,555
	Extremely Important	0.02	0.22	0.76	25,833

This table shows the correlation between workers' reported expertise in and importance of tasks in the main survey. The cells show the probability of reporting the column expertise score, conditional on reporting the row importance score. *Sample*: The table is based on all completed survey responses.

Table S8. Persistence of Importance Score, $P($Follow-Up$_{it}|$Main$_{it})$

		Follow Up					
		Not Important	Somewhat Important	Important	Very Important	Extremely Important	Observations
Main Survey	Not Important	0.70	0.14	0.09	0.05	0.03	5,713.00
	Somewhat Important	0.32	0.32	0.22	0.11	0.03	4,770.00
	Important	0.16	0.21	0.36	0.21	0.06	8,739.00
	Very Important	0.10	0.11	0.28	0.38	0.13	10,872.00
	Extremely Important	0.08	0.06	0.13	0.31	0.42	10,001.00

This table shows the correlation between the task importance scores reported in the follow-up and the main survey. Whereas the main survey asked about the tasks' general importance, the follow-up asked about their importance in the past two weeks. The cells show the probability of reporting the column importance score in the follow-up, conditional on having reported the row importance cell score in the main survey. *Sample*: The table is based on all individuals who completed the main and follow-up surveys.

Table S9. Persistence of Expertise Score, $P($Follow-Up$_{it}|$Main$_{it})$

		Follow Up			
		Low	Average	High	Observations
Main	Low	0.64	0.29	0.07	7,122.00
	Average	0.16	0.60	0.24	14,240.00
	High	0.04	0.23	0.72	17,781.00

This table shows the correlation between the task expertise scores reported in the follow-up and the main survey. The cells show the probability of reporting the column expertise score in the follow-up, conditional on having reported the row expertise score in the main survey. *Sample*: The table is based on all individuals who completed the main and follow-up surveys.

2. Adoption of ChatGPT

2.A. Market Share of ChatGPT. Table S10 presents estimates of the relative website traffic for the five most popular generative AI chatbots. The traffic estimates are sourced from `semrush.com` and include both desktop and mobile devices. ChatGPT maintains a market share of 80-90% in Denmark, the United States, and worldwide, with a slightly higher share in Denmark. Microsoft Copilot has recently gained market share from ChatGPT, particularly in the United States.

In November 2024, ChatGPT is estimated to receive 19 million visits from 2.3 million unique visitors in Denmark (38% of the population), 724 million visits from 100 million unique visitors in the United States (29% of the population), and 5,350 million visits from 676 million unique visitors worldwide (8% of the global population). Since November 2023, when our survey launched, the number of monthly visitors and visits to ChatGPT has increased by 23% and 95% in Denmark, 10% and 85% in the United States, and 32% and 110% worldwide.

Table S10. Website Traffic of Generative AI Chatbots

	November 2023 (Time of Survey)		November 2024 (Most Recent Data)	
	Share of Unique Visitors (%)	Share of Visits (%)	Share of Unique Visitors (%)	Share of Visits (%)
Panel A. Denmark				
ChatGPT	92.6	98.2	88.9	96.1
Gemini / Bard	6.6	1.5	5.4	0.9
Perplexity	0.5	0.3	1.5	0.5
Claude	0.2	0.1	1.7	1.4
Microsoft Copilot	0.1	0.0	2.5	1.0
Panel B. United States				
ChatGPT	90.0	93.5	81.1	90.9
Gemini / Bard	7.8	4.0	8.0	1.9
Perplexity	1.1	1.0	3.5	3.0
Claude	1.0	1.4	2.0	1.7
Microsoft Copilot	0.2	0.1	5.4	2.5
Panel C. Worldwide				
ChatGPT	88.6	92.8	81.5	91.0
Gemini / Bard	8.6	4.6	8.7	2.3
Perplexity	1.5	1.5	2.9	2.4
Claude	0.9	1.0	2.3	2.3
Microsoft Copilot	0.3	0.1	4.7	1.9

This table presents website traffic data (unique visitors and total visits) for the five most popular AI chatbots. The traffic estimates are sourced from `semrush.com` and include both desktop and mobile devices. Columns (1) and (2) display traffic shares as of November 2023, coinciding with the launch of our survey. Columns (3) and (4) present traffic data for November 2024, the most recent period available. The data was retrieved on November 20, 2024, and uses forecasts for the current month. Panels A, B, and C report website traffic for Denmark, the United States, and Worldwide, respectively. The websites analyzed are `chatgpt.com` and `openai.com/chatgpt` for ChatGPT, `gemini.google.com` and `bard.google.com` for Gemini/Bard, `perplexity.ai` for Perplexity, `claude.ai` for Claude, and `copilot.microsoft.com` for Microsoft Copilot.

2.B. Adoption across Occupations.

2.B.1. Domain of Use. In this section, we provide additional results on which domains ChatGPT is used in. Figure S2 shows that most workers who use ChatGPT for work also use it for leisure. Furthermore, workers are not more likely to use ChatGPT for "peripheral" tasks than "core" tasks. In particular, the task-level rates of adoption are 9.5% for peripheral tasks (that are "somewhat important" or "important") and 9.9% for core tasks (that are "very important" or "extremely important").

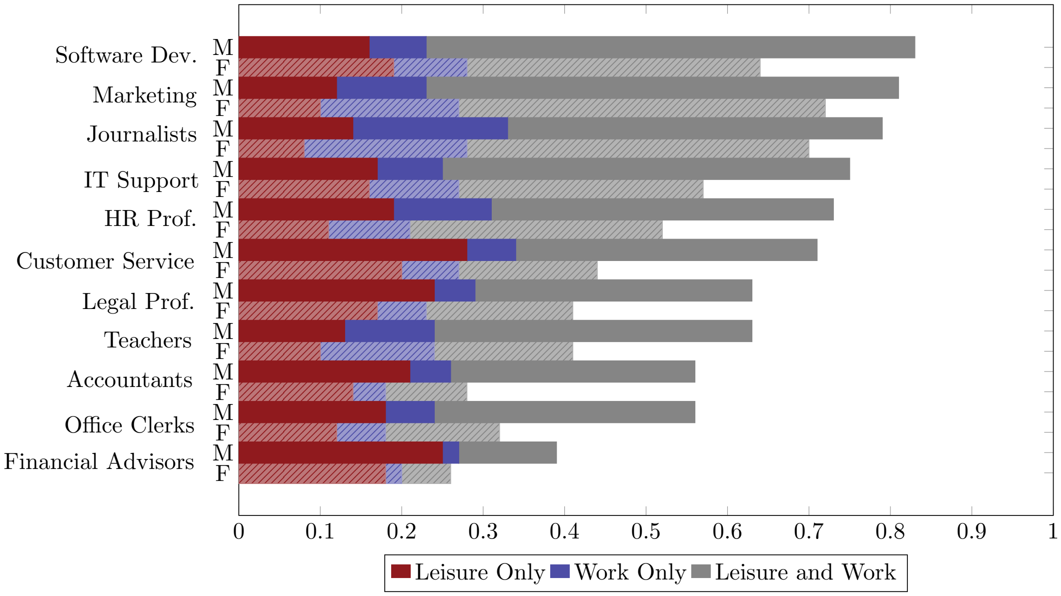

Fig. S2. Adoption of ChatGPT across Occupations. This figure shows the shares of male (M) and female (F) workers in each occupation who have used ChatGPT for leisure only, for work tasks only, or for both leisure and work. *Sample*: The figure is based on all completed survey responses.

2.B.2. Comparison to Other AI Surveys. In this section, we benchmark our rates of adoption with those found in other AI surveys.

ChatGPT Surveys. Our rates of adoption align with other smaller-scale national surveys of ChatGPT. In particular, using the (1) β-exposure scores to extrapolate outside our survey occupations (assuming a fixed ratio between exposure and adoption) implies 31% (22%) of workers in Denmark have used ChatGPT (for work). In comparison, (5) find that 35% of Danes had used ChatGPT by May 2024. Adoption rates for ChatGPT are similar in Denmark and the US and multiple times higher than for any other generative AI tool (5, 6).

ICT Surveys. Statistics Denmark conducts a nationally representative survey on "ICT Usage by Individuals", which provides some benchmarks for the gender gap in the adoption of ChatGPT. In 2023, all Danes used the internet (99% of both women and men), and there was no purpose for internet use with a similar gender gap as ChatGPT. Across all purposes and periods, the only statistic with a similar gender gap is "Downloading software (other than games software)" in 2008-2011, with women 20 percentage points less likely to do so (42% vs. 62%). However, while that gap is in the broad population, the gender gap we document exists within detailed job categories (e.g., comparing two software developers with similar task mixes).

Firm-Level Surveys. The adoption rates for ChatGPT are high relative to existing AI technologies measured in firm-based surveys. (7) document 3.2% of US firms used AI between 2016 and 2018, with adoption in the leading sector (Information) below 10%. (8) report 4.5% of Danish firms used AI tools in 2017. (9) measure five AI-embedded technologies, showing 5.8% of US firms used any of these in 2017. (10) show AI jobs constituted 0.8% of online vacancies in the US in 2018. (11) find 0.3% of employees at US public firms held AI-related positions in 2018.
The higher adoption rates for ChatGPT likely reflect that this is a worker-driven phenomenon. (12) document 5.4% of US firms reported using AI as of February 2024, with adoption in the leading sector (Information) around 20%. Our worker-level survey complements existing firm-based surveys by measuring workers' use of ChatGPT independent of firm-wide adoption initiatives.

2.C. Adoption within Occupations.

2.C.1. Educational Achievements. Table S11 shows that workers' adoption of ChatGPT is largely uncorrelated with their educational achievements as measured by their years of schooling (Panel a) or high school grades (Panel b).

Table S11. Educational Achievements and the Adoption of ChatGPT

(a) Years of Schooling

	Univariate						Multivariate	
	(1)	(2)	(3)	(4)	(5)	(6)	(7)	(8)
Years of Schooling	0.002						0.001	0.000
	(0.000)						(0.000)	(0.000)
Age (10 Years)		-0.068					-0.061	-0.047
		(0.003)					(0.005)	(0.007)
Experience (10 Years)			-0.123				-0.057	-0.021
			(0.009)				(0.010)	(0.015)
log(Earnings)				0.036			0.076	0.049
				(0.009)			(0.010)	(0.014)
Net Wealth / Earnings					-0.020		-0.003	-0.004
					(0.001)		(0.002)	(0.002)
Female						-0.161	-0.139	-0.116
						(0.008)	(0.008)	(0.011)
Occupation FE's	✓	✓	✓	✓	✓	✓	✓	✓
Workplace FE's								✓
Tasks FE's								✓
Mean of Outcome	0.372	0.372	0.372	0.372	0.372	0.372	0.372	0.372
Observations	15406	15406	15406	15406	15406	15406	15406	15406

(b) High School GPA (Cohorts Ages 22-38)

	Univariate							Multivariate	
	(1)	(2)	(3)	(4)	(5)	(6)	(7)	(8)	(9)
Years of Schooling	0.001							0.001	0.001
	(0.000)							(0.000)	(0.001)
GPA High School		0.002						0.007	-0.009
		(0.010)						(0.010)	(0.023)
Age (10 Years)			-0.049					-0.061	-0.075
			(0.018)					(0.021)	(0.048)
Experience (10 Years)				-0.023				0.035	0.035
				(0.027)				(0.031)	(0.082)
log(Earnings)					0.036			-0.020	-0.002
					(0.009)			(0.017)	(0.043)
Net Wealth / Earnings						-0.010		-0.008	-0.002
						(0.005)		(0.006)	(0.013)
Female							-0.217	-0.220	-0.201
							(0.018)	(0.018)	(0.040)
Occupation FE's	✓	✓	✓	✓	✓	✓	✓	✓	✓
Workplace FE's									✓
Tasks FE's									✓
Mean of Outcome	0.453	0.453	0.453	0.453	0.453	0.453	0.453	0.453	0.453
Observations	3079	3079	3079	3079	3079	3079	3079	3079	3079

This table compares workers within occupations and asks what characterizes those who have used ChatGPT for work. *Years of schooling* is the minimum years of schooling required for the workers' highest completed degree. *GPA High School* is the workers' grade point average in high school, standardized within cohorts. See the footnote to Table 1 in the main text for other variable definitions. Occupational fixed effects have been absorbed. Standard errors in parentheses. *Sample*: The table is based on all completed survey responses that can be linked to registry education data. Panel (a) focuses on all workers with a registered degree. Panel (b) focuses on workers for whom we have data on their high school diplomas, which generally cover cohorts aged 22-38.

2.C.2. Adoption Measures. Tables S12-S13 show that the gender and income gaps hold for a wide array of adoption measures. In particular, although the absolute gaps in Table S12 shrink for rare outcomes (e.g., active use for a core job task or Plus subscription), Table S13 shows that the relative effects of gender and income (measured in percent of the outcome mean) increase for these more intensive adoption measures.

Table S12. Who Has Adopted ChatGPT?

	Ever Used ChatGPT			Use Last Two Weeks			
	Used (1)	Used for Work (2)	Used for Core Task (3)	Use (4)	Use for Work (5)	Use for Core Task (6)	Plus Subscription (7)
Age (10 Years)	-0.095	-0.068	-0.043	-0.058	-0.051	-0.037	-0.010
	(0.005)	(0.004)	(0.004)	(0.004)	(0.004)	(0.004)	(0.002)
Experience (10 Years)	-0.072	-0.063	-0.052	-0.044	-0.036	-0.030	-0.010
	(0.010)	(0.010)	(0.009)	(0.009)	(0.009)	(0.008)	(0.005)
log(Earnings)	0.083	0.082	0.048	0.044	0.049	0.035	0.022
	(0.009)	(0.009)	(0.009)	(0.008)	(0.008)	(0.008)	(0.005)
Net Wealth / Earnings	0.001	-0.001	-0.002	-0.004	-0.003	-0.003	-0.003
	(0.002)	(0.002)	(0.002)	(0.002)	(0.001)	(0.001)	(0.001)
Female	-0.182	-0.138	-0.119	-0.138	-0.119	-0.100	-0.041
	(0.008)	(0.008)	(0.007)	(0.007)	(0.007)	(0.006)	(0.004)
Occupation FEs	✓	✓	✓	✓	✓	✓	✓
Mean of Outcome	0.552	0.398	0.244	0.323	0.271	0.183	0.061
Observations	17907	17907	15696	17907	17907	15696	17907

This table compares workers within occupations and asks what characterizes those who have adopted ChatGPT. The regressions control for occupation fixed effects. Standard errors in parentheses. The adoption measures are (1) "have used", (2) "have used for work", (3) "have used for a core task", (4) "Use last two weeks", (5) "use last two weeks for work", (6) "use last two weeks for a core task", and (7) "have an active Plus subscription". "Core tasks" are "very important" or "extremely important" to the worker's job. *Sample*: The figure is based on all completed survey responses. See the footnote to Table 1 in the main text for variable definitions. *Sample*: The table is based on all completed survey responses that can be linked to the registry data.

Table S13. Who Has Adopted ChatGPT? Relative Effects

	Ever Used ChatGPT			Use Last Two Weeks			
	Used (1)	Used for Work (2)	Used for Core Task (3)	Use (4)	Use for Work (5)	Use for Core Task (6)	Plus Subscription (7)
Age (10 Years)	-0.173	-0.170	-0.176	-0.179	-0.188	-0.201	-0.159
	(0.008)	(0.011)	(0.018)	(0.013)	(0.015)	(0.022)	(0.038)
Experience (10 Years)	-0.130	-0.159	-0.214	-0.138	-0.133	-0.166	-0.166
	(0.018)	(0.024)	(0.038)	(0.029)	(0.032)	(0.046)	(0.077)
log(Earnings)	0.150	0.206	0.198	0.137	0.180	0.189	0.353
	(0.016)	(0.022)	(0.036)	(0.026)	(0.029)	(0.042)	(0.079)
Net Wealth / Earnings	0.002	-0.002	-0.009	-0.011	-0.011	-0.014	-0.046
	(0.003)	(0.004)	(0.006)	(0.005)	(0.005)	(0.008)	(0.013)
Female	-0.331	-0.347	-0.488	-0.428	-0.439	-0.549	-0.666
	(0.014)	(0.019)	(0.030)	(0.023)	(0.026)	(0.036)	(0.060)
Occupation FEs	✓	✓	✓	✓	✓	✓	✓
Mean of Outcome	0.552	0.398	0.244	0.323	0.271	0.183	0.061
Observations	17907	17907	15696	17907	17907	15696	17907

This table compares workers within occupations and asks what characterizes those who have used ChatGPT. The outcome variables are normalized by their means, such that the estimates measure relative effects. The regressions control for occupation fixed effects. Standard errors in parentheses. See the footnote to Table 1 in the main text for variable definitions.

2.C.3. Controlling for Worker Beliefs. Table S14 investigates whether the disparities in the adoption of ChatGPT from Table 1 in the main text are related to differences in workers' beliefs about the technology.

Column (5) shows the estimated gender gap in adoption is unaffected by controlling for workers' assessed time savings from ChatGPT. Column (6) shows that controlling for the uncertainty of workers' beliefs shrinks the gender gap in adoption from 11.8 to 10.4 pp, reflecting women are more uncertain in their assessments of ChatGPT (Table 2, Column (2) of the main text).

Table S14. Who Has Used ChatGPT for Work? Controlling for Worker Beliefs

	(1)	(2)	(3)	(4)	(5)	(6)
Age	-0.006	-0.007	-0.006	-0.005	-0.005	-0.005
	(0.000)	(0.000)	(0.000)	(0.001)	(0.001)	(0.001)
Experience	-0.009	-0.006	-0.006	-0.003	-0.003	-0.003
	(0.001)	(0.001)	(0.001)	(0.001)	(0.001)	(0.001)
log(Earnings)	0.088	0.082	0.070	0.049	0.047	0.047
	(0.008)	(0.009)	(0.009)	(0.012)	(0.012)	(0.011)
Net Wealth / Earnings	-0.008	-0.001	-0.002	-0.003	-0.002	-0.001
	(0.002)	(0.002)	(0.002)	(0.002)	(0.002)	(0.002)
Female	-0.176	-0.138	-0.128	-0.118	-0.118	-0.104
	(0.007)	(0.008)	(0.008)	(0.010)	(0.010)	(0.010)
Occupation FEs		✓	✓	✓	✓	✓
Task Importance FEs			✓	✓	✓	✓
Workplace FEs				✓	✓	✓
Beliefs, Share of Exposed Tasks					✓	✓
Uncertainty						✓
Adj R^2	0.092	0.171	0.191	0.255	0.258	0.271
Observations	17907	17907	17907	17907	17907	17907

This table shows how gaps in the adoption of ChatGPT are affected by controlling for worker beliefs about the capabilities of ChatGPT. Column (1) reports the raw multivariate gaps pooling across our 11 exposed occupations. Column (2) adds occupation fixed effects, reflecting the specification in Table 1, Column (6) in the main text. Column (3) adds fixed effects for task importance levels (330 fixed effects, reflecting 5 importance levels for the 6 job tasks of each of the 11 occupations). Column (4) adds workplace fixed effects, reflecting the specification in Table 1, Column (7) in the main text. Column (5) controls for worker beliefs about the productivity of ChatGPT. Column (6) controls for the the uncertainty of workers' beliefs. *Sample*: The table is based on all completed survey responses.

3. Beliefs about ChatGPT

Table S15, Panel A shows how workers' beliefs about ChatGPT vary across occupations. Panel B shows how beliefs depend on workers' prior use of the tool.

Table S15. Worker Beliefs about ChatGPT

			Expertise Complementarity			Individual	Cross-Task Substitution		
	Productivity	Uncertain	Negative	Neutral	Positive	Productivity	Zero	Inelastic	Elastic
	(1)	(2)	(3)	(4)	(5)	(6)	(7)	(8)	(9)
Panel A. By Occupations									
Marketing	0.452	0.504	0.304	0.432	0.264	0.407	0.340	0.398	0.262
	(0.323)	(0.500)	(0.348)	(0.347)	(0.340)	(0.348)	(0.474)	(0.490)	(0.440)
Journalists	0.233	0.576	0.468	0.356	0.177	0.205	0.385	0.462	0.154
	(0.236)	(0.494)	(0.385)	(0.338)	(0.302)	(0.235)	(0.487)	(0.499)	(0.361)
Software Dev.	0.363	0.432	0.430	0.366	0.205	0.275	0.332	0.495	0.173
	(0.313)	(0.495)	(0.383)	(0.332)	(0.312)	(0.319)	(0.471)	(0.500)	(0.378)
IT Support	0.419	0.394	0.413	0.380	0.207	0.348	0.365	0.421	0.213
	(0.300)	(0.489)	(0.348)	(0.318)	(0.287)	(0.309)	(0.482)	(0.494)	(0.410)
HR Prof.	0.393	0.552	0.397	0.419	0.184	0.321	0.434	0.346	0.220
	(0.283)	(0.497)	(0.354)	(0.330)	(0.266)	(0.284)	(0.496)	(0.476)	(0.414)
Teachers	0.210	0.574	0.358	0.495	0.147	0.191	0.613	0.249	0.139
	(0.232)	(0.494)	(0.371)	(0.363)	(0.255)	(0.244)	(0.487)	(0.432)	(0.345)
Customer Service	0.380	0.378	0.377	0.411	0.212	0.349	0.351	0.312	0.337
	(0.330)	(0.485)	(0.361)	(0.338)	(0.280)	(0.344)	(0.477)	(0.464)	(0.473)
Legal Prof.	0.352	0.634	0.421	0.405	0.174	0.298	0.377	0.436	0.188
	(0.287)	(0.482)	(0.368)	(0.344)	(0.278)	(0.289)	(0.485)	(0.496)	(0.390)
Office Clerks	0.417	0.529	0.333	0.437	0.230	0.361	0.393	0.368	0.239
	(0.315)	(0.499)	(0.347)	(0.343)	(0.291)	(0.321)	(0.488)	(0.482)	(0.427)
Accountants	0.382	0.668	0.390	0.410	0.200	0.297	0.326	0.388	0.286
	(0.331)	(0.471)	(0.387)	(0.362)	(0.305)	(0.333)	(0.469)	(0.487)	(0.452)
Financial Adv.	0.466	0.611	0.369	0.468	0.164	0.433	0.240	0.400	0.361
	(0.342)	(0.487)	(0.370)	(0.358)	(0.271)	(0.371)	(0.427)	(0.490)	(0.480)
Panel B. By Prior Use									
Ever Used	0.381	0.450	0.378	0.409	0.213	0.326	0.346	0.420	0.234
	(0.315)	(0.498)	(0.365)	(0.340)	(0.306)	(0.325)	(0.476)	(0.494)	(0.424)
Never Used	0.355	0.641	0.399	0.426	0.175	0.306	0.419	0.345	0.236
	(0.306)	(0.480)	(0.373)	(0.352)	(0.271)	(0.313)	(0.493)	(0.475)	(0.425)
All Workers	0.370	0.532	0.387	0.416	0.197	0.317	0.377	0.388	0.235
	(0.311)	(0.499)	(0.369)	(0.345)	(0.292)	(0.320)	(0.485)	(0.487)	(0.424)

This table shows workers' beliefs about ChatGPT, split by occupation (Panel A) and prior use of ChatGPT (Panel B). Column (1) reports the share of job tasks where access to ChatGPT can halve working times for an *average* worker. Column (2) shows the share of tasks where workers are (very) uncertain about their productivity assessments (from Column (1)). Columns (3)-(5) show the share of job tasks in which ChatGPT delivers respectively smaller, similar, and larger time savings for workers with greater task expertise. Column (6) shows the share of job tasks where access to ChatGPT can halve workers' *own* working times. Column (7) shows the share of workers who will not complete more of a task if ChatGPT can save time in it. Column (8) is the share of workers who will complete more of a task but will not dedicate a larger share of their work time to the task. Column (9) is the share of workers who will dedicate a larger share of their time to a task if ChatGPT can save time completing it. *All workers* are averages with equal weights to each occupation. *Sample*: The table is based on all completed survey responses. Columns (6)-(9) focus on the control group as these survey questions come after the treatment page.

Figure S3 shows workers' stated reasons for why their time savings from ChatGPT are lower than the average worker's. Panel (a) splits workers by whether they have ever used ChatGPT before. Workers who have never used ChatGPT are 124% (6 pp) more likely to state that their time savings are lower because they do not know how to use the tool.

Panel (b) shows the reasons for male and female workers separately. Men are most likely to give their expertise levels as the reason they gain less from ChatGPT. By contrast, women are more concerned about the correctness of ChatGPT's responses (i.e., "hallucinations").

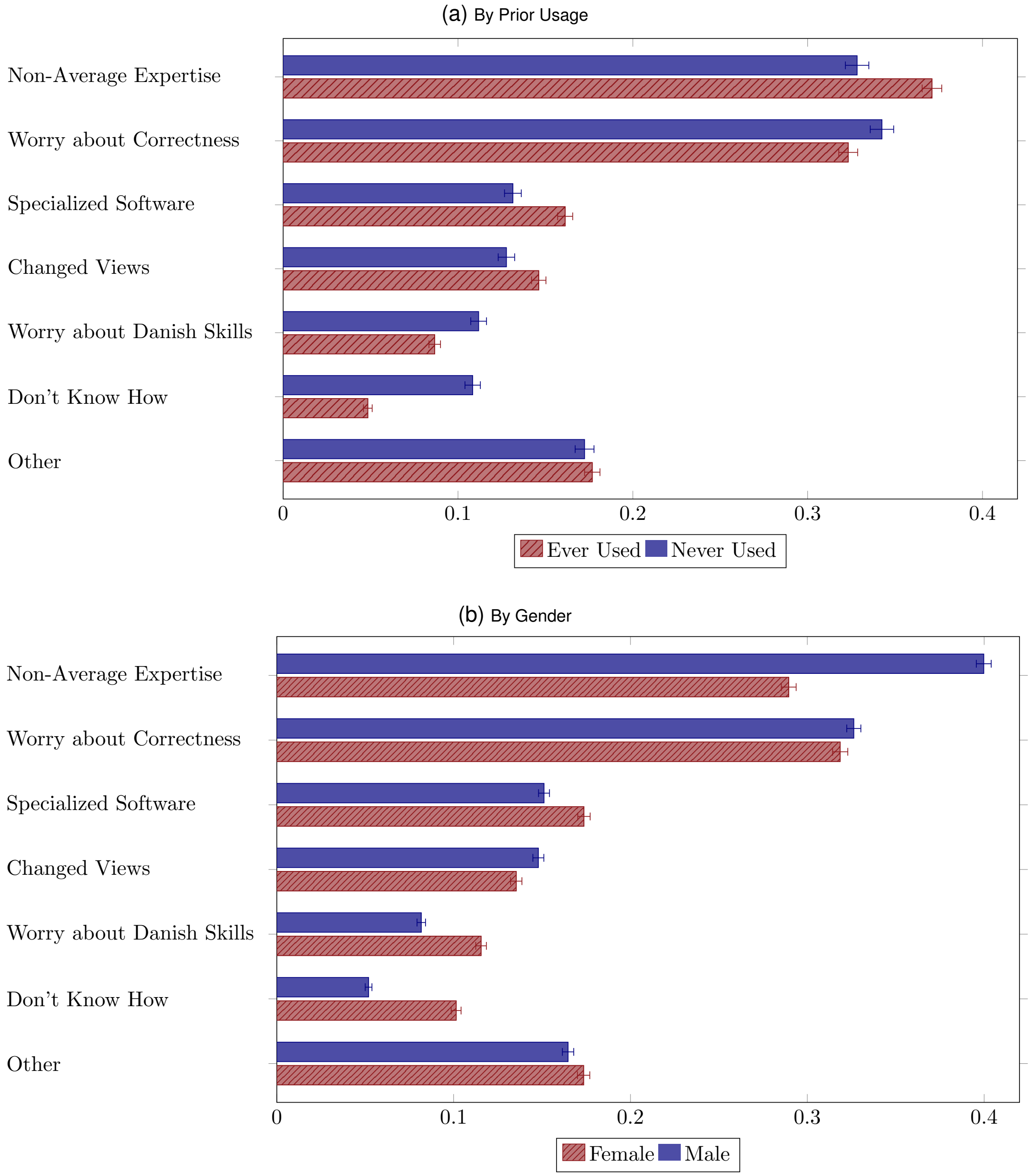

Fig. S3. Reasons Why Own Time Savings Are Smaller Than Average Worker's. This figure focuses on job tasks where workers state ChatGPT could halve the working times for an average worker but not for themselves. The bars report workers' reasons for why the time savings are smaller for themselves than for the average worker. The bars may sum to more than 100% as workers may report multiple reasons. Panel (a) splits workers by their prior usage of ChatGPT. Panel (b) splits workers by their gender. The figures assign each occupation with a uniform weight for each group, ensuring that any differences do not reflect differences in occupational compositions. *Sample*: The figure is based on all completed survey responses of the control group.

4. Beliefs versus Adoption

4.A. Prior Use, Beliefs, and Intended Use. Figure S4 shows the relationship between workers' prior use of ChatGPT, estimated time savings from the tool in their job tasks, and intended future use of ChatGPT in the tasks. The figure reveals several insights about how workers' use of ChatGPT relates to their beliefs about it.

First, workers see a substantial productivity potential in ChatGPT regardless of their actual experiences with the tool. Workers who have used ChatGPT estimate that it can halve their working times in 32.6% of their job tasks, whereas the estimated time savings are only slightly lower at 30.6% among workers who have never used it before. This suggests that the benefits of ChatGPT are widely perceived by workers and not limited to workers who have actually used the tool. Table S15 shows that workers who have used the tool are more certain in their assessment, however.

Second, workers who have not used ChatGPT at this point also do not intend to use it going forward. For example, even in the tasks where these "never users" say ChatGPT could halve their working times, only 3.3% intend to use it in the next two weeks. Their intended adoption is even lower (0.5%) in the job tasks where they do not see large time savings. This suggests that the adoption curve for ChatGPT has passed its inflection point and that few non-users are looking to start using it.

Third, even among workers who have used ChatGPT before and estimate it can half their working times, only 36.3% intend to use it in the relevant tasks within the next two weeks. Taking workers' state time savings at face value, this suggests large unrealized productivity gains from ChatGPT. In the next section, we investigate what prevents workers from using ChatGPT despite stating the tool could save time.

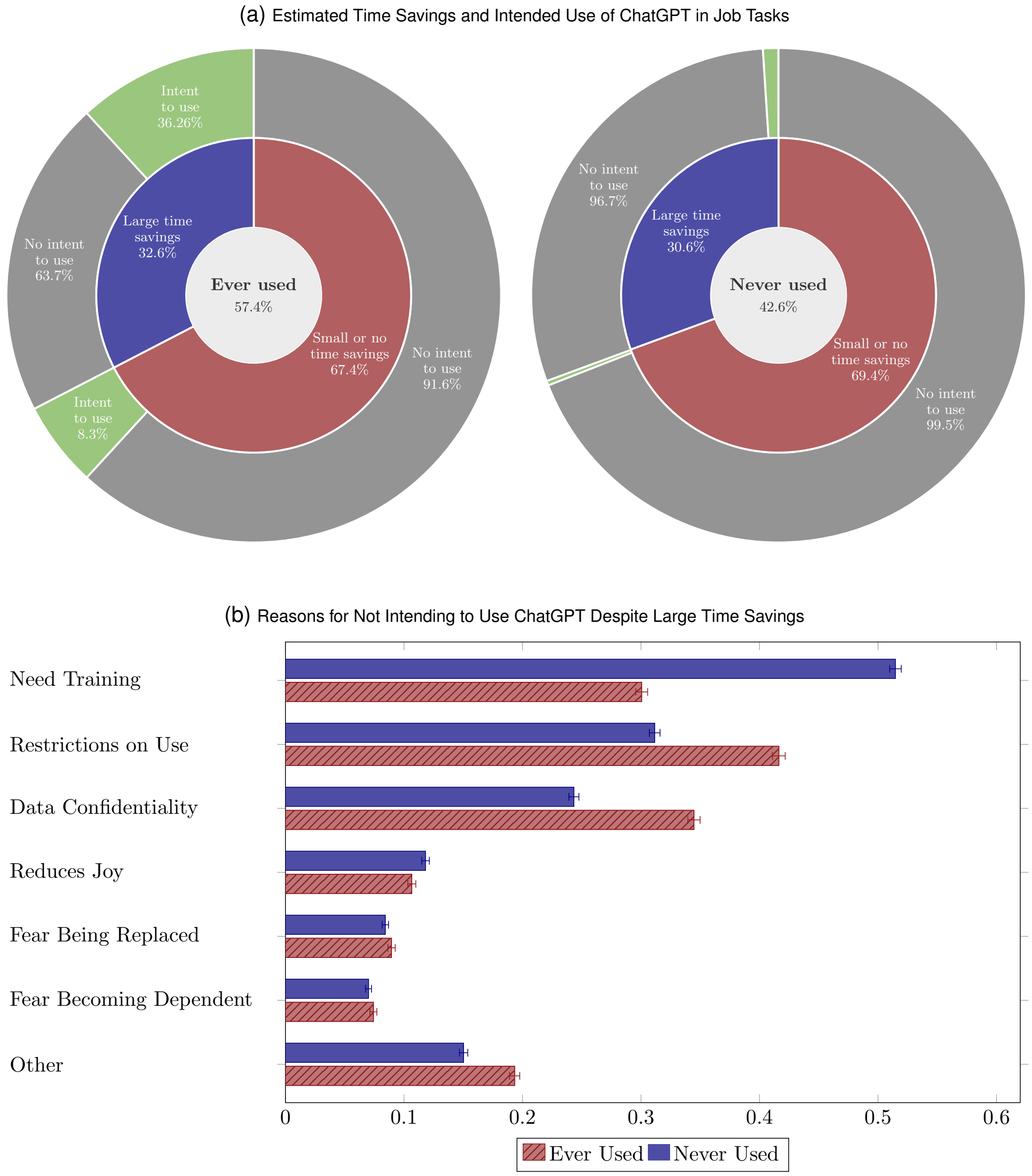

Fig. S4. Beliefs, Intended Use, and Frictions by Prior Experience with ChatGPT. This figure shows the relationship between workers' estimated time savings from ChatGPT, intended use of the tool, and adoption frictions in their job tasks. The figures are split by workers' prior usage of ChatGPT. The figure assigns each occupation with a uniform weight for each group, ensuring that any differences by prior usage do not reflect differences in occupational compositions. Panel (a) shows the relationship between workers' estimated time savings from the ChatGPT in their job tasks (inner ring) and their intended use of ChatGPT (in the coming two weeks) in the job tasks (outer ring). Panel (b) shows workers' reported reasons for not intending to use ChatGPT despite stating it could generate large time savings. The figure focuses on tasks workers expect to perform in the coming two weeks. The excluded job tasks (that workers do not expect to perform) represent 21% (21%) of the tasks with large reported time savings by workers who have ever (never) used ChatGPT. As workers may report multiple reasons for their friction, the reason bars may sum to more than 100%. Whiskers represent 95% confidence bands. *Sample*: The figure is based on all completed survey responses of the control group.

4.B. Adoption Frictions. Figure S4.(b) shows workers' reported reasons for not intending to use ChatGPT despite stating it could halve working times in a job task they perform. The figure studies the ("Large time savings" & "No intent to use") segments of Figure S4.(a), focusing on tasks that workers expect to encounter, and asking what prevents these workers from intending to use ChatGPT. The job tasks displayed in Figure D.1.(b) represent 43% (76%) of the tasks with large reported time savings by workers who have ever (never) used ChatGPT.

The most important frictions relate to firm policies: 42% of the workers report they need training to use ChatGPT, and 36% report employers actively restrict their usage.[‖] "Existential fears" of becoming redundant in the job or dependent on technology are the least important adoption frictions, with less than 9% of workers reporting these fears as reasons for not using ChatGPT. The relevant frictions to adoption differ by workers' experiences with the tool. 51% of workers who have not used ChatGPT

‖The need for training reflects workers who personally need training, as opposed to firms requiring training they cannot access. For example, the share of workers who state they need training is 45% among workers who do not face employer restrictions on use, as opposed to 36% among workers who face restrictions.

before state the need for training as their reason for not using ChatGPT, while 42% of workers who have tried the tool report employer restrictions as the reason for not using it.

The stated need for training may seem surprising since ChatGPT does not require special technical training. For example, (13) show that workers reap substantial time savings from ChatGPT after minimal training in the tool. Figure S4.(b) suggests that part of the need for training could reflect a barrier to trying it out, as the friction diminishes by 28% (12 pp) when focusing on workers who have actually tried the tool.[**] Still, even among workers who have used the tool and state it could help save time, 13% report the need for training as their reason for not using it on the job. This suggests that the "need for training" is not just about accessing the tool but also about how to integrate it into one's daily work life. Finally, the large share of workers who state they need training to use ChatGPT could reflect that workplace training is more institutionalized in Denmark (14). To investigate this hypothesis, Table S16 links our survey responses to an administrative register on course training in Denmark. Workers' reported need for training to use ChatGPT is largely uncorrelated with their workplace training rates in 2022, before the arrival of ChatGPT. Instead, the reported need for training is systematically higher among female, older, more experienced, and lower-earning workers. These findings indicate that the need for training to use ChatGPT is not just a Danish phenomenon.

Table S16. What Predicts Workers' Stated Need for Training?

	(1)	(2)
Workplace training rate	0.0461	-0.0245
	(0.0289)	(0.0290)
log(Earnings)		-0.0485***
		(0.0140)
Female		0.0479***
		(0.0131)
Age (10 Years)		0.0628***
		(0.0065)
Experience (10 Years)		0.0541**
		(0.0171)
Net Wealth / Earnings		-0.0001
		(0.0001)
Occupation FE's	✓	✓
Observations	6334	6334

This table asks what predicts workers' stated need for training. The sample focuses on job tasks where workers report large time savings but do not intend to use ChatGPT. The outcome variable is an indicator that takes one if workers report "It would require training before I can benefit from ChatGPT" as their reason for not using ChatGPT. *Workplace Training Rate* is defined as the share of coworkers (employed in the same occupation and workplace at the time of the survey) that participated in course training in 2022 (measured as any activity in the Course Participation Register (VEUV)). See Table 1 in the main text for definitions of the remaining variables. *Sample*: The table is based on all completed survey responses of the control group.

Table S17 shows that frictions to adoption helps explain the systematic differences between occupations in the use of ChatGPT (Fig. 1 of the main text). For example, while 82% of financial advisors face an adoption friction, 35% of software developers report the same. The relevant frictions also differ by occupation. Employer restrictions are more likely to bind in occupations that handle sensitive information, such as financial advisors and legal professionals. Less IT-prone occupations, such as teachers, report they need training to use ChatGPT, whereas this is less of a concern for software developers. Customer service representatives avoid ChatGPT due to fears of being replaced or becoming dependent on technology. Finally, in occupations where writing is a core competency, such as journalism and teaching, workers resist ChatGPT because it diminishes their enjoyment of their jobs.

[**] Similarly, in Figure S3.(a), 50% fewer state they "do not know how" to use ChatGPT as a reason their benefits from it are lower.

Table S17. Adoption Frictions by Occupations

Occupation	Friction (1)	Need Training (2)	Restrictions on Use (3)	Data Confidentiality (4)	Reduces Joy (5)	Fear Being Replaced (6)	Fear Becoming Dependent (7)	Other (8)
Marketing	.385	.347	.381	.374	.106	.088	.072	.158
Journalists	.482	.611	.152	.211	.235	.152	.105	.235
Software Dev.	.35	.253	.481	.269	.104	.066	.104	.2
IT Support	.479	.343	.405	.367	.11	.072	.106	.146
HR Prof.	.594	.435	.282	.289	.097	.048	.048	.264
Teachers	.609	.659	.05	.184	.237	.047	.119	.143
Customer Service	.491	.369	.163	.313	.214	.238	.112	.144
Legal Prof.	.67	.375	.464	.426	.071	.056	.052	.194
Office Clerks	.617	.454	.257	.259	.084	.073	.039	.232
Accountants	.647	.583	.318	.299	.071	.071	.067	.107
Financial Adv.	.817	.328	.61	.204	.067	.069	.049	.11

This table shows the adoption frictions by occupations. Column (1) reports the share of tasks where workers do not intend to use ChatGPT despite its ability to save time for them (that is, tasks subject to an "adoption friction"). The table focuses on tasks workers expect to perform. Columns (2)-(8) report the reasons (in shares) workers report for their adoption frictions. As workers may report multiple reasons for their friction, the shares may sum to more than 100%. *Sample*: The table is based on all completed survey responses of the control group.

Finally, it is worth noting that the analysis above focuses on workers' *intended* use, and individuals' intentions often fail to manifest in *actual* behaviors (15, 16). For example, workers may be habit-driven in their everyday work, even in the face of shocks that shift beliefs. Indeed, Table S18 shows that workers' reported time savings from ChatGPT are less correlated with their actual prior use (Column 2) than their intended use (Column 3) in their job tasks. Overall, the task-level correlation between estimated time savings and usage is 0.27 for intended use and 0.19 for prior use.

Table S18. Time Savings, Prior Use, and Intended Use of ChatGPT in Job Tasks

		Share of Tasks (1)	Prior Use (2)	Intended Use (3)
Male	Large time savings	.317	.233	.278
	Small or no time savings	.683	.078	.062
Female	Large time savings	.327	.126	.169
	Small or no time savings	.673	.037	.033

This table shows the relationship between workers' estimated time savings and usage of ChatGPT in their job tasks. Column (1) reports the share of job tasks with "large" versus "small or no" time savings from ChatGPT. Column (2) reports the share of these job tasks where workers have used ChatGPT, and Column (3) shows the share where they intend to use ChatGPT. *Sample*: The table is based on all completed survey responses of the control group.

4.C. Information Treatment. This section studies the causal role of worker beliefs in the adoption of ChatGPT. In particular, we expose a random set of workers to the expert assessment of ChatGPT described in Section 1.A. Using this experiment, we study whether information shifts workers' perception of ChatGPT and, if so, whether the changed beliefs affect their adoption decisions. We preregistered our experiment at AEA-RCT-R-0012527, with the analysis in this section focusing on the productivity treatment.

4.C.1. Experiment. We randomize workers into treatment and control groups. The treatment group see the expert assessments and short explanations from Sections 1.A.5-1.A.6. The control group sees a summary of their assessments. Figure S5 displays an example of the treatment and control pages in the survey. Table S19 shows that worker characteristics, assessments, and adoption (before treatment) balance across the treatment and control groups.

(a) Treatment

Write commentaries, columns, or scripts	
**Question:** Can ChatGPT save time?	
Your assessment	Small or no time savings
Expert assessment	Large time savings
Explanation of the **expert assessment**	ChatGPT can generate drafts, suggest changes, and provide ideas for articles, etc.
**Question:** Can someone with greater expertise save more time?	
Your assessment	Similar time savings for the journalist with greater expertise

(b) Control

Write commentaries, columns, or scripts	
**Question:** Can ChatGPT save time?	
Your assessment	Small or no time savings
**Question:** Can someone with greater expertise save more time?	
Your assessment	Similar time savings for the journalist with greater expertise

Fig. S5. Treatment Page of the Information Experiment. This figure shows an English translation of the treatment screen of the information experiment, focusing on an example task of journalists. Panel (a) shows the treatment page, comparing expert assessments of the time savings from ChatGPT with workers' prior assessments. The expert assessments are supported by short explanations. Panel (b) shows the placebo page of the control group, summarizing workers' prior assessments.

Table S19. Balance Table for the Information Experiment

	Control (1)	Treatment - Control (2)	p-value (3)
Panel A: Characteristics			
Age	45.40	0.15	0.45
		(0.20)	
log(Earnings)	13.11	0.02	0.02
		(0.01)	
Experience	7.12	0.05	0.47
		(0.08)	
Net Wealth/Earnings	4.10	-1.05	0.23
		(0.88)	
Female	0.49	-0.00	0.59
		(0.01)	
Panel B: Adoption			
Used	0.55	-0.01	0.17
		(0.01)	
Used for Work	0.40	-0.01	0.17
		(0.01)	
Used for Core Task	0.21	-0.01	0.19
		(0.01)	
Panel C: Beliefs			
Productivity	0.36	-0.00	0.63
		(0.01)	
Negative Complementarity	0.38	-0.01	0.37
		(0.01)	
Neutral Complementarity	0.42	-0.00	0.92
		(0.01)	
Positive Complementarity	0.19	0.01	0.21
		(0.01)	
Observations	6,077	6,016	

This table shows worker characteristics, assessments, and adoption (before treatment) balance across the treatment arms. *Sample*: The table is based on all completed survey responses in the *productivity treatment* and *control* arms that can be linked to registry data.

4.C.2. Identification Strategy. Our identification strategy combines the information treatment with workers' pre-treatment deviations from the expert assessments. Following (17), the idea is that workers who are initially far off from the expert assessments receive a larger information treatment from exposure to the information.

Our outcomes of interest Y_i^{Post} are workers' posterior beliefs and adoption behaviors. The reduced-form estimating equation reads

$$Y_i^{\text{Post}} = \beta_0 + \beta_1 \text{Deviation}_i^{\text{Pre}} + \beta_2 \text{Treated}_i + \beta_3 \text{Treated}_i \times \text{Deviation}_i^{\text{Pre}} + \epsilon_i, \qquad [3]$$

where $\text{Deviation}_i^{\text{Pre}}$ is worker i's pre-treatment deviation from the expert assessments, and Treated_i indicates the information treatment. We denote superscripts by pre- and post-treatment timing.

Our coefficient of interest in Equation Eq. (3) is β_3, which measures the effect of the treatment by workers' initial deviations from the expert assessments. We expect a negative β_3 if the information shifts beliefs, such that workers who initially underestimate the productivity of ChatGPT update their beliefs positively and adopt the tool accordingly.

To estimate effects at the worker level, we use workers' average deviations across their six surveyed job tasks, $\text{Deviation}_i^{\text{Pre}} = \frac{1}{6}\sum_{t=1}^{6}\left(\text{Productivity}_{it}^{\text{Pre}} - \text{Productivity}_t^{\text{Expert}}\right)$. The productivity beliefs are measured on a 0-1 scale, such that β_3 measures the effect of information for workers who initially underestimate all six job tasks.

4.C.3. Main Results. Table S20, Panel A shows the impact of the information treatment on workers' posterior beliefs and adoption behaviors. Column (1) reports the impact on workers' beliefs about their own time savings from ChatGPT. The information treatment is successful in shifting workers' beliefs, with the deviations from the expert assessments shrinking by 14.9%.[††]

Columns (2)-(3) study workers' adoption behaviors in the main survey, reporting effects on workers' intended use and interest in information about use cases of ChatGPT. Overall, the information treatment has muted effects on adoption, with magnitudes that are about 10%-20% of the effect on beliefs and not statistically significant.

[††] The treatment also has a direct negative impact on worker beliefs β_2. This could reflect that workers perceive technology experts as overoptimistic about the productivity of ChatGPT.

Columns (4)-(5) report impacts in the follow-up survey. Workers' beliefs remain shifted two weeks after the treatment, with about 55%-85% of the original effect persisting. However, workers are not more likely to have actually used ChatGPT in the two weeks that followed the treatment.

Table S20. Effects of the Information Treatment on Beliefs and Adoption Behaviors

	Main Survey			Follow Up	
	Ind. Productivity (1)	Ind. Productivity Follow Up Sample (2)	Intent to Use in Job Tasks (3)	Individual Productivity (4)	Use in Job Tasks (5)
Panel A: All					
Deviation × Treated	-0.149	-0.156	-0.014	-0.087	0.015
	(0.012)	(0.020)	(0.013)	(0.024)	(0.019)
Treated	-0.021	-0.021	-0.007	-0.014	0.006
	(0.005)	(0.008)	(0.004)	(0.010)	(0.007)
Deviation	0.735	0.761	0.181	0.544	0.104
	(0.009)	(0.015)	(0.010)	(0.019)	(0.015)
Control Means	0.309	0.300	0.104	0.265	0.079
Observations	12093	4051	12092	4051	4051
Panel B: Male					
Deviation × Treated	-0.092	-0.084	0.003	-0.086	0.034
	(0.016)	(0.026)	(0.019)	(0.030)	(0.028)
Treated	-0.004	-0.001	-0.001	-0.015	0.013
	(0.007)	(0.012)	(0.007)	(0.014)	(0.012)
Deviation	0.739	0.745	0.241	0.581	0.148
	(0.013)	(0.021)	(0.015)	(0.025)	(0.023)
Control Means	0.312	0.296	0.146	0.264	0.108
Observations	6124	2189	6124	2189	2189
Panel C: Female					
Deviation × Treated	-0.218	-0.255	-0.033	-0.082	-0.005
	(0.020)	(0.032)	(0.015)	(0.041)	(0.023)
Treated	-0.034	-0.038	-0.013	-0.010	0.000
	(0.007)	(0.011)	(0.005)	(0.014)	(0.007)
Deviation	0.733	0.783	0.112	0.493	0.044
	(0.014)	(0.023)	(0.012)	(0.031)	(0.015)
Control Means	0.307	0.305	0.061	0.266	0.045
Observations	5969	1862	5968	1862	1862

This table shows the reduced-form effects of the information treatment on workers' posterior beliefs and adoption behaviors. Equation Eq. (3) provides the regression specification. Columns (1)-(3) show the effects on workers' beliefs and intended use (coming two weeks) in the main survey. Columns (4)-(5) show effects on workers' beliefs and actual use (past two weeks) in the follow-up survey. *Deviation* is workers' average deviation from the expert assessments of the productivity of ChatGPT in their surveyed job tasks; see Section 4.C.2 for the definition. *Individual Productivity* is workers' average individual productivity of ChatGPT in their surveyed job tasks. Occupation fixed effects are absorbed. Standard errors are in parentheses. *Sample*: The table is based on all completed survey responses in the *productivity treatment* and *control* arms.

4.C.4. Adoption Frictions. The muted effects of information on the adoption of ChatGPT are consistent with the frictions documented in Section 4.B of the main text. In particular, Table S20 shows the correlation β_1 between workers' prior beliefs ("Deviation") and adoption behaviors ("Intent to Use") is also muted and around $\beta_1 \approx 20\%$. In fact, our reduced-form estimates on intended use are somewhat smaller than but within the confidence bands of those predicted by the correlations.[‡‡] Furthermore, Table S21 shows workers' responses to the information treatment are hindered by the same barriers as those reported in the general population in Figure 4.(b) of the main text, namely "needing training" and "employer restrictions."

Table S21. Adoption Frictions in the Information Experiment

	Need Training (1)	Restrictions on Use (2)	Data Confidentiality (3)	Reduces Joy (4)	Fear Being Replaced (5)	Fear Becoming Dependent (6)	Other (7)
Panel A: Male							
Complier	0.439	0.429	0.414	0.082	0.096	0.032	0.163
	(0.063)	(0.063)	(0.060)	(0.041)	(0.033)	(0.035)	(0.046)
Control	0.374	0.413	0.314	0.105	0.082	0.084	0.146
	(0.009)	(0.009)	(0.009)	(0.006)	(0.005)	(0.005)	(0.007)
Observations	36744	36744	36744	36744	36744	36744	36744
Panel B: Female							
Complier	0.497	0.237	0.197	0.119	0.020	0.045	0.268
	(0.037)	(0.035)	(0.034)	(0.023)	(0.019)	(0.018)	(0.030)
Control	0.481	0.299	0.263	0.112	0.065	0.060	0.189
	(0.008)	(0.008)	(0.007)	(0.005)	(0.004)	(0.004)	(0.007)
Observations	35814	35814	35814	35814	35814	35814	35814

This table shows complier and control means for the reported adoption frictions, that is, reasons workers do not use ChatGPT despite stating it can halve their working times in tasks they expect to perform. Compliers are workers who report adoption friction only if receiving the treatment. Section 5.B describes the econometric specification. *Sample*: The table is based on all completed survey responses in the *productivity treatment* and *control* arms.

4.C.5. Gender Gaps. In Panels B and C of Table S20, we split our experiment by gender. Women's beliefs are more than twice as responsive to the treatment on impact, as their deviations from the expert assessments shrink by 22% in the main survey, compared with 9% for men. Women are also more likely to intend to use ChatGPT following the treatment, whereas men's adoption behaviors are unaltered by the information. Women's larger initial belief response dissipates entirely after two weeks, however, as the treatment effects on workers' beliefs shrink to 8%. The effects on women's actual use two weeks after are also attenuated and not statistically significant.

In summary, these findings show the gender gap in adoption does *not* reflect women are less responsive to information about the technology. On the contrary, women respond more to the information but face barriers that prevent their further adoption. In particular, Table S21, Panel B confirms the "need for training" is actively hindering more women from taking advantage of ChatGPT.

[‡‡] The effects predicted by the correlation is given by $\beta_1^{\text{Adoption}} \times \beta_3^{\text{Ind.Prod.}}$ (i.e., the correlation between adoption and beliefs, scaled by the reduced-form impacts on beliefs).

5. Econometric Specifications

5.A. Sample Selection Correction using Randomized Incentives. This section describes our procedure for controlling for selection into the survey based on worker unobservables. Following (3), we specify a sample selection model that exploits the exogenous variation in survey response rates from our randomized participation incentives.

Individual i responds to the survey if his incentive Z_i exceeds his latent resistance U_i:

$$R_i = \mathbf{1}[p(Z_i) \geq U_i], \tag{4}$$

where $p(Z)$ is the participation propensity score among individuals with incentive level Z, and we normalize the distribution of resistances to be uniform, $U_i \sim U[0, 1]$.[§§]

Let Y_i^* denote the outcome of interest for individual i. We assume a linear Marginal Survey Response (MSR) function:

$$Y_i^* = \alpha_0 + \alpha_1 U_i. \tag{5}$$

Combining Equations Eq. (4)-Eq. (5), the average response in our survey among individuals with incentive Z is

$$\mathbb{E}[Y|Z] = \frac{1}{p(Z)} \int_0^{p(Z)} [\alpha_0 + \alpha_1 U] dU = \alpha_0 + \frac{\alpha_1}{2} p(Z). \tag{6}$$

Our parameter of interest is the average survey response (ASR) in the general population:

$$\text{ASR} = E[Y^*] = \int_0^1 [\alpha_0 + \alpha_1 U] dU = \alpha_0 + \frac{\alpha_1}{2}. \tag{7}$$

We can estimate the ASR by first calculating the propensity scores for each incentive level $P(Z)$, then estimating the linear regression Eq. (6), and finally plugging the estimated MSR coefficients $(\hat{\alpha}_0, \hat{\alpha}_1)$ into Equation Eq. (7).

As is common in the MTE literature (see, e.g., (22, 23)), we estimate the ASR in the support of the propensity scores:

$$\text{ASR}(p_{min}, p_{max}) = E[Y^* | p_{min} \leq U_i \leq p_{max}] \tag{8}$$

$$= \frac{1}{(p_{max} - p_{min})} \int_{p_{min}}^{p_{max}} [\alpha_0 + \alpha_1 U] dU = \alpha_0 + \frac{\alpha_1}{2} \frac{p_{max}^2 - p_{min}^2}{p_{max} - p_{min}}, \tag{9}$$

which avoids extrapolations outside our domain of identification. Table S2, Column (3) reports our estimates of Equation Eq. (9).

5.B. Adoption Frictions in the Information Experiment. This section describes how we estimate the frictions that hinder the information treatment from affecting workers' adoption behaviors. Let *friction compliers* denote workers who face an adoption friction (i.e., do not intend to use ChatGPT despite believing it can save time) if they receive the information treatment. We profile the friction reasons (e.g., employer restrictions, required training, etc.) faced by these compliers by running the 2SLS regressions at the worker-task level:

$$\text{Friction}_{it}^{\text{Post}} = \beta_{10} + \beta_{11} \text{Deviation}_{it}^{\text{Pre}} + \beta_{12} \text{Treated}_i + \beta_{13} \text{Treated}_i \times \text{Deviation}_{it}^{\text{Pre}} + \epsilon_{1it} \tag{10}$$

$$\text{Reason}_{it}^{\text{Post}} = \beta_{20} + \beta_{21} \text{Deviation}_{it}^{\text{Pre}} + \beta_{22} \widehat{\text{Friction}}_{it}^{\text{Post}} + \epsilon_{2it}, \tag{11}$$

where $\text{Friction}_{it}^{\text{Post}}$ indicates that worker i faces an adoption friction in task t, $\text{Reason}_{it}^{\text{Post}}$ denotes the reason for the friction, $\text{Deviation}_{it}^{\text{Pre}}$ is worker i's pre-treatment deviation from the expert assessments in task t, and Treated_i indicates the information treatment. Equation Eq. (10) is the first stage, and the 2SLS estimate of β_{22} identifies the share of friction compliers with the particular reason for their friction, as reported in Table S21. The specification in Equations Eq. (10)-Eq. (11) follows (24).

[§§] (3) develop a general framework for correcting for sample selection that allows for multiple dimensions of unobserved heterogeneity. Because we only use the explicitly randomized variation from the participation incentives, our selection correction procedure falls into the more traditional class of single threshold models (18, 19). (20, 21) provide an overview of selection models and lay out the Marginal Treatment Effects (MTE) framework.

6. Job Tasks in the Survey

This section lists the job tasks we include for each occupation in the survey. Table S22 provides the English translations and Table S23 is the original Danish versions.

Table S22. Job Tasks Included in the Survey (English Translation)

Occupation	Job Task
Accountants & Auditors	Prepare detailed reports on audit findings.
Accountants & Auditors	Supervise auditing of establishments, and determine scope of investigation required.
Accountants & Auditors	Examine and evaluate financial and information systems, recommending controls to ensure system reliability and data integrity.
Accountants & Auditors	Confer with company officials about financial and regulatory matters.
Accountants & Auditors	Prepare, examine, or analyze accounting records, financial statements, or other financial reports to assess accuracy, completeness, and conformance to reporting and procedural standards.
Accountants & Auditors	Compute taxes owed and prepare tax returns, ensuring compliance with payment, reporting, or other tax requirements.
Customer Service Rep.	Confer with customers by telephone or in person to provide information about products or services, take or enter orders, cancel accounts, or obtain details of complaints.
Customer Service Rep.	Keep records of customer interactions or transactions, recording details of inquiries, complaints, or comments, as well as actions taken.
Customer Service Rep.	Check to ensure that appropriate changes were made to resolve customers' problems.
Customer Service Rep.	Contact customers to respond to inquiries or to notify them of claim investigation results or any planned adjustments.
Customer Service Rep.	Determine charges for services requested, collect deposits or payments, or arrange for billing.
Customer Service Rep.	Review claims adjustments with dealers, examining parts claimed to be defective, and approving or disapproving dealers' claims.
Financial Advisors	Interview clients to determine their current income, expenses, insurance coverage, tax status, financial objectives, risk tolerance, or other information needed to develop a financial plan.
Financial Advisors	Recommend to clients strategies in cash management, insurance coverage, investment planning, or other areas to help them achieve their financial goals.
Financial Advisors	Manage client portfolios, keeping client plans up-to-date.
Financial Advisors	Implement financial planning recommendations, or refer clients to someone who can assist them with plan implementation.
Financial Advisors	Analyze financial information obtained from clients to determine strategies for meeting clients' financial objectives.
Financial Advisors	Answer clients' questions about the purposes and details of financial plans and strategies.
HR Professionals	Interpret and explain human resources policies, procedures, laws, standards, or regulations.
HR Professionals	Hire employees and process hiring-related paperwork.
HR Professionals	Inform job applicants of details such as duties and responsibilities, compensation, benefits, schedules, working conditions, or promotion opportunities.
HR Professionals	Prepare or maintain employment records related to events, such as hiring, termination, leaves, transfers, or promotions, using human resources management system software.
HR Professionals	Address employee relations issues, such as harassment allegations, work complaints, or other employee concerns.
HR Professionals	Schedule or conduct new employee orientations.
IT Support	Answer user inquiries regarding computer software or hardware operation to resolve problems.
IT Support	Oversee the daily performance of computer systems.
IT Support	Read technical manuals, confer with users, or conduct computer diagnostics to investigate and resolve problems or to provide technical assistance and support.
IT Support	Set up equipment for employee use, performing or ensuring proper installation of cables, operating systems, or appropriate software.
IT Support	Enter commands and observe system functioning to verify correct operations and detect errors.
IT Support	Maintain records of daily data communication transactions, problems and remedial actions taken, or installation activities.
Journalists	Write commentaries, columns, or scripts.
Journalists	Coordinate and serve as an anchor on news broadcast programs.
Journalists	Examine news items of local, national, and international significance to determine topics to address, or obtain assignments from editorial staff members.
Journalists	Analyze and interpret news and information received from various sources to broadcast the information.
Journalists	Arrange interviews with people who can provide information about a story.
Journalists	Present news stories, and introduce in-depth videotaped segments or live transmissions from on-the-scene reporters.
Legal Professionals	Prepare affidavits or other documents, such as legal correspondence, and organize and maintain documents in paper or electronic filing system.
Legal Professionals	Prepare legal documents, including briefs, pleadings, appeals, wills, contracts, and real estate closing statements.
Legal Professionals	Prepare for trial by performing tasks such as organizing exhibits.
Legal Professionals	Investigate facts and law of cases and search pertinent sources, such as public records and internet sources, to determine causes of action and to prepare cases.
Legal Professionals	Meet with clients and other professionals to discuss details of case.
Legal Professionals	File pleadings with court clerk.
Marketing Professionals	Prepare reports of findings, illustrating data graphically and translating complex findings into written text.
Marketing Professionals	Collect and analyze data on customer demographics, preferences, needs, and buying habits to identify potential markets and factors affecting product demand.
Marketing Professionals	Conduct research on consumer opinions and marketing strategies, collaborating with marketing professionals, statisticians, pollsters, and other professionals.
Marketing Professionals	Measure and assess customer and employee satisfaction.
Marketing Professionals	Measure the effectiveness of marketing, advertising, and communications programs and strategies.
Marketing Professionals	Attend staff conferences to provide management with information and proposals concerning the promotion, distribution, design, and pricing of company products or services.
Office Clerks	Operate office machines, such as computers, voice mail systems, photocopiers, and scanners.
Office Clerks	Answer telephones, direct calls, and take messages.
Office Clerks	Communicate with customers, employees, and other individuals to answer questions, disseminate or explain information, take orders, and address complaints.
Office Clerks	Compile, copy, sort, and file records of office activities, business transactions, and other activities.
Office Clerks	Open, sort, and route incoming mail, answer correspondence, and prepare outgoing mail.
Office Clerks	Compute, record, and proofread data and other information, such as records or reports.
Software Developers	Write, analyze, review, and rewrite programs, using workflow chart and diagram, and applying knowledge of computer capabilities, subject matter, and symbolic logic.
Software Developers	Correct errors by making appropriate changes and rechecking the program to ensure that the desired results are produced.
Software Developers	Perform or direct revision, repair, or expansion of existing programs to increase operating efficiency or adapt to new requirements.
Software Developers	Consult with managerial, engineering, and technical personnel to clarify program intent, identify problems, and suggest changes.
Software Developers	Conduct trial runs of programs and software applications to be sure they will produce the desired information and that the instructions are correct.
Software Developers	Consult with and assist computer operators or system analysts to define and resolve problems in running computer programs.
Teachers	Prepare students for later grades by encouraging them to explore learning opportunities and to persevere with challenging tasks.
Teachers	Adapt teaching methods and instructional materials to meet students' varying needs and interests.
Teachers	Establish and enforce rules for behavior and procedures for maintaining order among students.
Teachers	Prepare objectives and outlines for courses of study, following curriculum guidelines or requirements of states and schools.
Teachers	Prepare, administer, and grade tests and assignments to evaluate students' progress.
Teachers	Prepare materials and classrooms for class activities.

Table S23. Job Tasks Included in the Survey (Original Danish Version)

Faggruppe	Arbejdsopgave
Revisions- og regnskabsmedarbejder	Udarbejde detaljerede revisionsrapporter.
Revisions- og regnskabsmedarbejder	Overvåge revision af virksomheder og fastsætte omfanget af en sådan undersøgelse.
Revisions- og regnskabsmedarbejder	Undersøge finansielle systemer eller IT-systemer og anbefale kontroller af systemers pålidighed og dataintegritet.
Revisions- og regnskabsmedarbejder	Rådføre sig med virksomhedsledelsen om økonomiske og regulatoriske forhold.
Revisions- og regnskabsmedarbejder	Forberede, undersøge eller analysere regnskabsdokumenter eller andre finansielle rapporter for at vurdere nøjagtighed, fuldstændighed samt overholdelse af rapporterings- og proceduremæssige standarder.
Revisions- og regnskabsmedarbejder	Beregne skatteforpligtelser og udarbejde selvangivelser i overensstemmelse med skatteregler.
Kundesupport	Tale med kunder personligt eller telefonisk for at informere om produkter eller tjenester, modtage ordrer, opsige konti eller indhente detaljer om kundeklager.
Kundesupport	Registrere kontakt eller transaktioner med kunder vedrørende detaljer om forespørgsler, klager eller kommentarer samt besluttede foranstaltninger.
Kundesupport	Kontrollere at passende foranstaltninger blev foretaget for at løse kunders problemer.
Kundesupport	Kontakte kunder for at besvare forespørgsler eller informere dem om udfaldet af klageundersøgelser eller planlagte foranstaltninger.
Kundesupport	Fastlægge pris på efterspurgte ydelser, udstede fakturaer, eller indsamle deposita og betalinger.
Kundesupport	Gennemgå reklamationer med forhandlere, undersøge hævdede fejl i produkter, og godkende eller afvise forhandleres krav.
Økonomisk rådgiver	Interviewe klienter for at afgøre deres nuværende indkomst, udgifter, forsikringsdækning, skattestatus, økonomiske mål, risikotolerance eller andre oplysninger, der er nødvendige for at udvikle en økonomisk plan.
Økonomisk rådgiver	Anbefale klienter strategier inden for likviditetsstyring, forsikringsdækning, investeringsplanlægning eller andre områder for at hjælpe dem med at opnå deres økonomiske mål.
Økonomisk rådgiver	Forvalte klientporteføljer og holde klientplaner opdaterede.
Økonomisk rådgiver	Gennemføre anbefalinger fra en økonomisk plan eller henvise klienter til nogen, der kan hjælpe dem med implementeringen.
Økonomisk rådgiver	Analysere økonomiske oplysninger om klienter for at fastlægge strategier, der opfylder klienters økonomiske mål.
Økonomisk rådgiver	Besvare klienters spørgsmål om formålet for og detaljerne i økonomiske planer og strategier.
HR-medarbejder	Fortolke og forklare politikker, procedurer, love, standarder eller reguleringer inden for HR-området.
HR-medarbejder	Ansætte medarbejdere og behandle ansættelsesrelateret papirarbejde.
HR-medarbejder	Informere jobansøgere om ansættelsesvilkår, såsom jobindhold og -ansvar, løn og goder, arbejdstider og -forhold samt muligheder for forfremmelse.
HR-medarbejder	Opbygge eller vedligeholde ansættelsesregistre i forbindelse med ansættelse, fratrædelse, orlov, omplacering eller forfremmelser ved hjælp af HR-software.
HR-medarbejder	Håndtere medarbejderrelationer, herunder sager om chikane, arbejdsrelaterede klager eller andre bekymringer fra medarbejdere.
HR-medarbejder	Planlægge eller gennemføre introduktioner for nye medarbejdere.
IT-supporter	Besvare brugerhenvendelser vedrørende drift af computerhardware eller -software for at løse problemer.
IT-supporter	Overvåge daglig performance af computersystemer.
IT-supporter	Læse tekniske manualer, kommunikere med brugere eller udføre computerdiagnostik for at undersøge og løse problemer eller yde teknisk hjælp og support.
IT-supporter	Opsætte udstyr til medarbejdere og udføre eller sikre korrekt installation af kabler, operativsystemer eller software.
IT-supporter	Indtaste kommandoer og observere computersystemers funktion for at bekræfte korrekt drift eller registrere fejl.
IT-supporter	Føre log over daglige dataoverførsler, installationer og tekniske problemer samt afhjælpende foranstaltninger.
Journalist	Skrive kommentarer, klummer eller artikler.
Journalist	Være vært på og tilrettelægge nyhedsudsendelser.
Journalist	Gennemgå nyheder af lokal, national og international betydning for at fastlægge hvilke emner, der skal behandles eller få tildelt historier fra redaktionen.
Journalist	Analysere og fortolke nyheder og information fra forskellige kilder for at videreformidle informationen.
Journalist	Arrangere interviews med personer, der kan give information om en historie.
Journalist	Præsentere nyhedshistorier og introducere dybdegående videosegmenter eller live transmissioner fra journalister på stedet.
Juridisk medarbejder	Forberede erklæringer eller andre dokumenter såsom juridisk korrespondance, organisere og vedligeholde dokumenter i et papirbaseret eller elektronisk arkivsystem.
Juridisk medarbejder	Forberede juridiske dokumenter, herunder processkrifter, forsvars- og anklageskrifter, appeller, testamenter, kontrakter og ejendomshandelsdokumenter.
Juridisk medarbejder	Forberede en retssag såsom at udarbejde bevismateriale.
Juridisk medarbejder	Undersøge fakta, omstændigheder og lovgivning i sager og søge relevante kilder, såsom offentlige registre og internetkilder, for at afklare årsager til søgsmål og forberede sager.
Juridisk medarbejder	Møde klienter og fagfolk for at drøfte sagsdetaljer.
Juridisk medarbejder	Indsende processkrifter til retssekretæren.
Marketingmedarbejder	Udarbejde rapporter, der illustrerer data grafisk og formidler komplekse sammenhænge i tekst.
Marketingmedarbejder	Indsamle og analysere data om kundeoplysninger, præferencer, behov og købsvaner for at identificere potentielle markeder og faktorer, der påvirker produkters efterspørgsel.
Marketingmedarbejder	Undersøge forbrugerholdninger og marketingstrategier i samarbejde med marketingmedarbejdere, statistikere, meningsmålere og andre fagfolk.
Marketingmedarbejder	Måle og vurdere kunde- og medarbejdertilfredshed.
Marketingmedarbejder	Måle effekten af marketing, reklame og kommunikationsindsatser og -strategier.
Marketingmedarbejder	Deltage i medarbejdermøder for at give information og forslag vedrørende markedsføring, distribution, design og prissætning af virksomhedens produkter eller tjenester.
Kontoransat eller sekretær	Betjene kontormaskiner såsom computere, telefonsvarere, kopimaskiner og scannere.
Kontoransat eller sekretær	Besvare telefonopkald, viderestille opkald og modtage beskeder.
Kontoransat eller sekretær	Kommunikere med kunder, brugere, medarbejdere og andre for at besvare spørgsmål, formidle eller forklare information, modtage ordrer og håndtere klager.
Kontoransat eller sekretær	Udarbejde, sortere og arkivere optegnelser over kontor-, forretnings- og andre aktiviteter.
Kontoransat eller sekretær	Åbne, sortere og videresende indgående post, besvare henvendelser og forberede udgående post.
Kontoransat eller sekretær	Bearbejde, registrere og tjekke data og anden information såsom optegnelser eller rapporter.
Softwareudvikler	Skrive, analysere, gennemgå og ændre programmer, fx. ved hjælp af rutediagrammer samt ved anvendelse af viden om emnet, computers kapacitet og symbolsk logik.
Softwareudvikler	Rette fejl i programmer og kontrollere, at ønskede resultater opnås.
Softwareudvikler	Udføre eller lede revision, fejlretning eller udvidelse af eksisterende programmer for at øge driftseffektiviteten eller møde nye krav.
Softwareudvikler	Konsultere ledelses-, ingeniør- og teknisk personale for at afklare målet for et computerprogram, identificere problemer og foreslå ændringer.
Softwareudvikler	Teste programmer og softwareapplikationer for at sikre, at de genererer det ønskede output og at instruktionerne er korrekte.
Softwareudvikler	Konsultere og hjælpe IT-teknikere eller systemanalytikere med at identificere og løse problemer i forbindelse med kørsel af computerprogrammer.
Lærer	Forberede eleverne til senere klassetrin ved at motivere dem til at udforske læringsmuligheder og støtte dem i at løse udfordrende opgaver.
Lærer	Tilpasse undervisningsmetoder og -materialer for at imødekomme elevers forskellige behov og interesser.
Lærer	Fastlægge og håndhæve regler for adfærd og procedurer for at opretholde orden blandt eleverne.
Lærer	Forberede undervisningsmål og -forløb i overensstemmelse med læseplaner eller krav fra stat, kommune eller skole.
Lærer	Formulere, afholde og bedømme prøver og opgaver for at vurdere elevers udvikling.
Lærer	Indrette klasseværelser og fysiske materialer til undervisningsaktiviteter.

7. Invitation Letter

This section contains the invitation letter for the main survey. We sent three reminders, two by e-mail (Digital Post) and one by text (SMS).

The English translation starts on page 28, with the original Danish version on page 30.

301

November 2023

Artificial intelligence and your job tasks

Dear [name]

Statistics Denmark is inviting you to participate in a research project about ChatGPT and your job tasks. You participate by clicking the link below and answering the questionnaire.

ChatGPT is a chatbot with artificial intelligence. You have been selected because you work in an occupation where it may be relevant to use ChatGPT.

Your answers are important regardless of your knowledge of artificial intelligence or ChatGPT. Your participation will advance research about new technology in the labor market. Everyone who completes the questionnaire will automatically participate in a lottery with **a prize of [X,XXX] Kr. tax free**.

Statistics Denmark is conducting the survey for researchers at the University of Copenhagen and the University of Chicago. It takes **about 15 minutes** to complete the questionnaire.

Start the survey [url]

Or access www.dst.dk/ditsvar and enter your response code **[code]**.

Statistics Denmark handles your data confidentially. We convey the results in a way that makes it impossible to see how individuals have responses, and the data is used solely for statistical and scientific purposes.

Participation is voluntary. If you do not wish to participate, you can indicate this: [refusal_link]

If you have questions, you can write to info@dstsurvey.dk or call on 7777 7708 (every day between 9am and 4pm). Please provide your response code when contacting us.

Best regards,

Marie Fuglsang
Head of Division, DST Survey

Anders Humlum
Assistant Professor, University of Chicago

302

We take care of your answers

Statistics Denmark processes personal data in accordance with the rules of the European General Data Protection Regulation (GDPR) and the Danish Data Protection Act. Furthermore, Statistics Denmark has a data confidentiality policy, which ensures that information about citizens is protected and exclusively used for statistical or scientific studies. We handle your responses confidentially and only use the results in such a way that no one can see your individual answers.

Your responses in this survey are exclusively used for statistical and scientific purposes within this survey. Your responses are deleted or archived according to applicable legislation when the information no longer serves a purpose in the study.

Statistics Denmark is the data processor for collecting your responses in the survey. Once you have answered the questions, your responses in pseudonymized form are forwarded to the University of Copenhagen, which is the data controller for the survey. This means that your responses cannot be directly traced back to you.

The legal basis for data processing is Article 6(1)(e) of the GDPR. If sensitive information is involved, the legal basis is Article 9(2)(j) of the GDPR and § 10 of the Danish Data Protection Act.

You can contact the data protection advisor of Statistics Denmark via databeskyttelse@dst.dk

Read more:

Statistics Denmark's compliance with GDPR also applies to the information about you in this survey. Read more about the processing and what rights and complaint options you have here

Read more about security and confidentiality at Statistics Denmark here

Read more about the data controller here

Who is invited to Statistics Denmark's surveys?

Anyone residing in Denmark can be invited to participate in one of Statistics Denmark's surveys. In our surveys, it is important to know the opinions and attitudes of the entire population across gender, age, education, and residence.

Why may we contact you?

Statistics Denmark has three main tasks according to the Statistics Denmark Act:

- to collect, process, and publish statistical information about society, possibly in co-operation with other statistics producers. In addition, to prepare statistical analyses and forecasts.
- to contribute to international statistical cooperation.
- to perform statistical projects for private and public customers for a fee under the rules of income-generating activities.

It is as part of the third bullet that we are allowed to contact you about this survey.

Invitation Letter – Danish Version

303

November 2023

Kunstig intelligens og dine arbejdsopgaver

Kære [navn]

Danmarks Statistik inviterer dig til at deltage i et forskningsprojekt om ChatGPT og dine arbejdsopgaver. Du deltager ved at klikke på nedenstående link og svare på spørgeskemaet.

ChatGPT er en chatbot med kunstig intelligens. Du er blevet udvalgt, fordi du arbejder i et erhverv, hvor det kan være relevant at bruge ChatGPT.

Dine svar er vigtige uanset dit kendskab til kunstig intelligens eller ChatGPT. Din deltagelse vil fremme forskning i ny teknologi på arbejdsmarkedet. Alle der gennemfører spørgeskemaet, deltager automatisk i lodtrækningen om **en præmie på [X.XXX] kr. skattefrit.**

Danmarks Statistik gennemfører spørgeskemaet for forskere på Københavns Universitet og University of Chicago. Det tager **ca. 15 minutter** at besvare spørgeskemaet.

Start undersøgelsen [url]

Eller gå ind på www.dst.dk/ditsvar og tast svarkoden **[kode]**

Danmarks Statistik behandler dine svar fortroligt. Vi formidler resultaterne på en måde, så ingen kan se, hvad den enkelte har svaret og data anvendes alene til statistiske og videnskabelige formål.

Det er frivilligt at deltage. Ønsker du ikke at deltage, kan du tilkendegive det: [refusal_link]

Har du spørgsmål, kan du skrive til info@dstsurvey.dk eller ringe på tlf. 7777 7708 (alle dage ml. kl. 9-16). Oplys venligst din svarkode ved henvendelse.

Med venlig hilsen

Marie Fuglsang
Kontorchef, DST Survey

Anders Humlum
Adjunkt, University of Chicago

304

Vi passer på dine svar

Danmarks Statistik behandler personoplysninger i overensstemmelse med reglerne i den europæiske databeskyttelsesforordning (GDPR) og den danske databeskyttelseslov. Danmarks Statistik har derudover en datafortrolighedspolitik, som sikrer, at oplysninger om borgerne beskyttes og udelukkende behandles til statistiske eller videnskabelige undersøgelser. Vi behandler dine svar fortroligt og bruger kun resultaterne på en måde, så ingen kan se, hvad du har svaret.

Dine svar i denne undersøgelse bruges udelukkende til statistiske og videnskabelige formål i denne undersøgelse. Dine svar slettes eller arkiveres efter gældende lovgivning, når oplysningerne ikke længere har et formål i undersøgelsen.

Danmarks Statistik er databehandler for indsamlingen af dine svar i undersøgelsen. Når du har svaret på spørgsmålene, videregives dine svar i pseudonymiseret form til Københavns Universitet, der er dataansvarlig for undersøgelsen. Det betyder, at dine svar ikke direkte kan tilbageføres til dig.

Retsgrundlaget for databehandling er databeskyttelsesforordningens artikel 6, stk. 1, litra e. Hvis der indgår følsomme oplysninger er retsgrundlaget forordningens artikel 9, stk. 2, litra j, og databeskyttelseslovens § 10.

Du kan kontakte Danmarks Statistiks databeskyttelsesrådgiver via databeskyttelse@dst.dk

Læs mere:

Danmarks Statistiks efterlevelse af GDPR gælder også for oplysningerne om dig i denne undersøgelse. Læs mere om behandlingen og hvilke rettigheder og klagemuligheder du har her

Læs mere om sikkerhed og fortrolighed hos Danmarks Statistik her

Læs mere om den dataansvarlige her

Hvem bliver inviteret til Danmarks Statistiks undersøgelser?

Alle, der har bopæl i Danmark, har mulighed for at blive inviteret til at deltage i en af Danmarks Statistiks undersøgelser. I vores undersøgelser er det vigtigt at kende meninger og holdninger fra hele befolkningen på tværs af køn, alder, uddannelse og bopæl.

Hvorfor må vi kontakte dig?

Danmarks Statistik har tre hovedopgaver ifølge Lov om Danmarks Statistik:

- at indsamle, bearbejde og offentliggøre statistiske oplysninger om samfundet, evt. i samarbejde med andre statistikproducenter. Herudover at udarbejde statistiske analyser og prognoser.
- at bidrage til det internationale statistiksamarbejde.
- at udføre statistiske opgaver for private og offentlige kunder mod betaling efter reglerne for indtægtsdækket virksomhed.

Det er som led i den tredje bullit, at vi har lov til at kontakte dig om denne undersøgelse.

8. Survey Questionnaire

This section contains our survey questionnaire. The questionnaire follows a common structure for the different occupations but with job tasks and titles tailored to each specific occupation.

For the sake of brevity, the questionnaire below focuses on one occupation (journalism), listing one of their six job tasks (write commentaries, columns, or scripts). We indicate the occupation-specific fields by square brackets.

The questionnaire below corresponds to the main survey. The follow-up survey follows the same structure with two exceptions: in Block 1, we ask about adoption and task importance in the past two weeks (Questions 3, 6, 8). We make this change to be consistent with the time window of intended adoption in Block 4 of the main survey (Questions 18 and 19). Second, we exclude Block 3 (Question 17, i.e., treatment/control) from the follow-up survey.

The English translation starts on page 33, with the original Danish version on page 43.

1. Introduction

ChatGPT is a chatbot that uses artificial intelligence. You have been selected to participate in this survey because you work in an occupation where it may be relevant to use ChatGPT.
Your participation is important regardless of your knowledge of artificial intelligence or ChatGPT.

Block 1: Adoption

2.a Occupation

Are you employed in [journalism]?

- Yes
- No

2.b Occupation [if 2.a='No']

Are you employed in one of the following occupations?
If you are employed in more than one occupation, please select your primary work area.

- Auditing and accounting
- Customer support
- Financial advising
- Human resources
- IT support
- Legal work
- Marketing
- Office and secretarial work
- Software development
- Teaching
- I am not employed in the above occupations

2.c Screen Out [if 2.b = 'I am not employed in the above occupations']

Thank you for participating in the survey. Unfortunately, you are not in the target group for this survey, and we therefore have no more questions.

3. Task Importance [all tasks]

We will first ask about some typical tasks among [journalists].
For each task, please assess how **important the task is for your job**.
Extremely important means that the task is critical for carrying out your job.
[Write commentaries, columns, or scripts]

- Not important
- Somewhat important
- Important
- Very important
- Extremely important

316 **4. Task Expertise** [all tasks]

For each task, please assess your **own expertise** in the task.

Expertise may, for example, come from previous experience with or innate abilities in performing the task.

[Write commentaries, columns, or scripts]

- Low expertise
- Average expertise
- High expertise

5. Awareness of ChatGPT

We will now ask about your experiences with ChatGPT.

Had you heard about ChatGPT before this survey?

- Yes
- No

6. Prior Use of ChatGPT [if 5='Yes']

Have you used ChatGPT?

- Yes
- No

7. Purposes of Prior Use [if 6='Yes']

For what purposes have you used ChatGPT?

- Work only
- Leisure only
- Both work and leisure

8. Prior Use in Job Tasks [if 7='Work only' or 7='Both work and leisure'; all tasks]

Have you used ChatGPT to perform the following job tasks?

Mark all tasks where you have used ChatGPT.

[Write commentaries, columns, or scripts]

9. Current Use of ChatGPT [if 6='Yes']

Have you used ChatGPT in the past two weeks?

- Yes
- No

10. Plus Subscription [if 6='Yes']

Do you have an active Plus subscription to ChatGPT?

- Yes
- No

317 **Block 2: Prior Beliefs**

11. Prior Beliefs: Productivity Introduction

Time Savings from ChatGPT

We will next ask for your assessment of whether ChatGPT can save time on various job tasks.
Note: Your answers are important regardless of your knowledge of ChatGPT. If you are not familiar with ChatGPT, we ask you to give your best guess. You will later get the opportunity to indicate how certain you are in your evaluations.

12. Prior Beliefs: Productivity of ChatGPT [all tasks]

Think of **a [journalist] with an average level of experience and expertise** trying to complete a given task. The worker has access to ChatGPT, the internet, a computer with existing software, and other tools typically used to complete the task.

Specify the following tasks according to the description below. Equivalent quality means someone reviewing the work would not be able to tell whether the worker completed it with or without assistance from ChatGPT.

Large time savings from ChatGPT

Specify the task's time savings as “Large” if **access to ChatGPT *can halve the time*** it takes for an average [journalist] to complete the task with equivalent quality.

Small or no time savings from ChatGPT

Specify the task's time savings as “Small or no” if **access to ChatGPT *cannot* halve the time** it takes for an average [journalist] to complete the task with equivalent quality.

Please provide your best estimates even if you are unsure of them.

[Write commentaries, columns, or scripts]

- Small or no time savings from ChatGPT
- Large time savings from ChatGPT

13. Uncertainty of Productivity Prior

How certain are you about your previous assessments of the time savings from ChatGPT for an average [journalist]?

- Very uncertain
- Uncertain
- Certain
- Very certain

318 **14. Prior Beliefs: Complementarity Introduction**

We now ask you to assess how the potential time savings from ChatGPT relate to [journalists'] expertise in given job tasks.

15. Prior Beliefs: Expertise Complementarity of ChatGPT [all tasks]

Imagine two [journalists] with average levels of experience and expertise but who differ in their expertise in a given task.

A [journalist] with greater expertise in the task: The worker has extensive experience in the task, has in-depth knowledge of its nuances, and has a track record of accuracy and efficiency in executing it.

A [journalist] with less expertise in the task: The worker has a broad understanding of the principles of the task but lacks expertise in executing the specific task.

The two [journalists] are similar in all other aspects except their expertise in the specific task.

Specify the following tasks according to whether access to ChatGPT in the task yields smaller, similar, or larger time savings for the worker with greater expertise compared to the worker with less expertise in the task.

Please provide your best estimates even if you are unsure of them.

[Write commentaries, columns, or scripts]

- Smaller time savings for the [journalist] with greater expertise
- Similar time savings for the [journalist] with greater expertise
- Larger time savings for the [journalist] with greater expertise

16. Uncertainty of Complementarity Prior

How certain are you about your previous assessments of how the time savings from ChatGPT relate to [journalists'] expertise?

- Very uncertain
- Uncertain
- Certain
- Very certain

Block 3: Treatment

17.a Productivity Treatment [if randomized into productivity treatment group; all tasks]

We previously asked you to evaluate the time savings from ChatGPT in various job tasks.

The University of Pennsylvania and OpenAI (the developer of ChatGPT) conducted an expert assessment in August 2023 of the time savings from ChatGPT in the same tasks. Researchers from the University of Copenhagen have validated and extended the expert assessments in collaboration with industry experts from Denmark.

Please take the time to review the table, as the information may become useful in the rest of the survey.

Note: You can continue by clicking on the "next" button after 15 seconds on this page. Once you proceed, you cannot go back to this table.

[Write commentaries, columns, or scripts]	
**Question:** Can ChatGPT save time?	
Your assessment	Small or no time savings
Expert assessment	Large time savings
Explanation of the **expert assessment**	ChatGPT can generate drafts, suggest changes, and provide ideas for articles, etc.
**Question:** Can someone with greater expertise save more time?	
Your assessment	Similar time savings for the [journalist] with greater expertise

17.b Complementarity Treatment [if randomized into complementarity treatment group; all tasks]
We previously asked you to evaluate the time savings from ChatGPT in various job tasks.

The University of Pennsylvania and OpenAI (the developer of ChatGPT) conducted an expert assessment in August 2023 of the time savings from ChatGPT in the same tasks. Researchers from the University of Copenhagen have validated and extended the expert assessments in collaboration with industry experts from Denmark.

Please take the time to review the table, as the information may become useful in the rest of the survey.

Note: You can continue by clicking on the "next" button after 15 seconds on this page. Once you proceed, you cannot go back to this table.

[Write commentaries, columns, or scripts]	
**Question:** Can ChatGPT save time?	
Your assessment	Small or no time savings
**Question:** Can someone with greater expertise save more time?	
Your assessment	Similar time savings for the [journalist] with greater expertise
Expert assessment	Smaller time savings for the [journalist] with greater expertise
Explanation of the **expert assessment**	A competent [journalist] can more easily prepare drafts and revise articles, etc., and therefore has less benefit from ChatGPT in the task.

321 **17.c Control** [if randomized into control group; all tasks]
We previously asked you to evaluate the time savings from ChatGPT in various job tasks.

Please take the time to review the table, as the information may become useful in the rest of the survey.

Note: You can continue by clicking on the "next" button after 15 seconds on this page. Once you proceed, you cannot go back to this table.

[Write commentaries, columns, or scripts]	
**Question:** Can ChatGPT save time?	
Your assessment	Small or no time savings
**Question:** Can someone with greater expertise save more time?	
Your assessment	Similar time savings for the [journalist] with greater expertise

322 **Block 4: Intended Adoption and Posterior Beliefs**

18. Intentions to Use ChatGPT

Do you expect to use ChatGPT in the next two weeks?

- Yes
- No

19. Intentions to Use in Job Tasks [if 18='Yes'; all tasks]

Do you expect to use ChatGPT in the following job tasks in the next two weeks?
[Write commentaries, columns, or scripts]

- Yes
- No

20. Posterior Beliefs: Individual Productivity of ChatGPT [all tasks]

Time savings from ChatGPT in your own job

Now, consider your own job, given your individual experience and expertise as a [journalist]. Assume that you have access to ChatGPT, the internet, a computer with existing software, and other tools you typically use to complete the task.

Specify the following tasks according to the description below. Equivalent quality means someone reviewing the work would not be able to tell whether you have completed it with or without assistance from ChatGPT.

Large time savings from ChatGPT

Specify the task's time savings as "Large" if **access to ChatGPT *can halve the time*** it takes for you to complete the task with equivalent quality.

Small or no time savings from ChatGPT

Specify the task's time savings as "Small or no" if **access to ChatGPT *cannot* halve the time** it takes for you to complete the task with equivalent quality.

Please provide your best estimates even if you are unsure of them.

[Write commentaries, columns, or scripts]

- Small or no time savings from ChatGPT
- Large time savings from ChatGPT

 Anders Humlum, Emilie Vestergaard

21. Uncertainty of Individual Productivity Prior

How certain are you about your previous assessments of the time savings from ChatGPT for yourself?

- Very uncertain
- Uncertain
- Certain
- Very certain

22. Final Questions: Introduction

We will now ask you some follow-up questions about your assessment of ChatGPT in the job tasks.

23.a Task Substitution

If ChatGPT saves time in completing a task, do you then expect to complete more of that type of tasks during your workday?

Please provide your best estimate, even if you are unsure.

- Yes
- No

23.b Task Substitution [if 23.a='Yes']

If ChatGPT saves time in completing a task, do you expect that type of tasks to occupy more of your workday, while other kinds of tasks occupy less?

The task can occupy more if the larger number of tasks completed outweighs the time saved in each individual task solution.

- Yes
- No

Block 5: Frictions

24. Beliefs Frictions [tasks with 12!=20]

Your assessment of the time savings from ChatGPT for an average [journalist] and yourself differed in the following job tasks.

Please indicate for each of the tasks the reasons why your assessments differed from one another.

[Write commentaries, columns, or scripts]

- I changed my view on the time savings from ChatGPT.
- The time savings for an average [journalist] are not relevant given my expertise.
- I don't know how to use ChatGPT.
- I use specialized software that does integrate with ChatGPT.
- I am concerned about the correctness of ChatGPT's responses.
- I am concerned about ChatGPT's lack of capabilities in Danish.
- Other, please specify: [open text field]

324 **S25: Adoption Frictions** [tasks with 19=’No’ and 20=’Large time savings from ChatGPT’]

You indicated for the following job tasks that:

1. ChatGPT can offer you a large time savings in completing the task.
2. You do not expect to use ChatGPT for the task in the next two weeks.

Please state the reasons why you do not expect to use ChatGPT in the job task despite its time savings.

[Write commentaries, columns, or scripts]

- I do not expect to encounter the task in the next two weeks.
- I am subject to restrictions on using ChatGPT in my job.
- I am concerned about how ChatGPT will handle my data confidentially.
- It would require training before I can benefit from ChatGPT.
- I fear that ChatGPT will eventually make me redundant in my job.
- ChatGPT will reduce my joy of performing the task.
- I am concerned about becoming dependent on ChatGPT in the task.
- Other, please specify: [open text field]

26. Information Sheets

Are you interested in receiving additional material with examples of how a [journalist] can use ChatGPT? The material has been prepared by researchers from the University of Copenhagen and the University of Chicago in collaboration with industry experts in Denmark. You can access the material at the end of the survey.

- Yes
- No

Side 27.(a) End of Survey [if 26=’Yes’]

Thank you for participating in the survey.

You can download the material prepared by researchers from the University of Copenhagen and the University of Chicago in collaboration with Danish industry experts, here: **ChatGPT for [journalists]**

If you win one of the prizes, you will be notified directly in your e-Boks.

We may contact you again in two weeks with a short follow-up survey. We hope very much that you are willing to participate in this brief follow-up.

Side 27.(b) End of Survey [if 26=’No’]

Thank you for participating in the survey.

If you win one of the prizes, you will be notified directly in your e-Boks.

We may contact you again in two weeks with a short follow-up survey. We hope very much that you are willing to participate in this brief follow-up.

1. Introduction

ChatGPT er en chatbot, der bruger kunstig intelligens. Du er blevet udvalgt til at deltage i denne undersøgelse, fordi du arbejder i et erhverv, hvor det kan være relevant at bruge ChatGPT.
Din deltagelse er vigtig uanset dit kendskab til kunstig intelligens eller ChatGPT.

Block 1: Adoption

2.a Occupation

Er du beskæftiget med [journalistik]?

- Ja
- Nej

2.b Occupation [if 2.a='Nej']

Er du beskæftiget inden for et af følgende områder?
Hvis du er beskæftiget indenfor flere områder, vælg da dit primære arbejdsområde.

- HR-arbejde
- IT-support
- Kontor- og sekretærarbejde
- Kundesupport
- Juridisk arbejde
- Marketing
- Revisions- og regnskabsarbejde
- Softwareudvikling
- Undervisning
- Økonomisk rådgivning
- Jeg er ikke beskæftiget inden for ovenstående arbejdsområder

2.c Screen Out [if 2.b = 'Jeg er ikke beskæftiget inden for ovenstående arbejdsområder']

Mange tak for at deltage i undersøgelsen.
Du er desværre ikke i målgruppen for undersøgelsen, og vi har derfor ikke flere spørgsmål.

3. Task Importance [all tasks]

Vi vil først spørge ind til nogle typiske arbejdsopgaver blandt [journalister].
Til hver opgave bedes du vurdere, hvor **vigtig opgaven er for dit arbejde**.
Ekstremt vigtig betyder, at opgaven er kritisk for varetagelsen af dit nuværende job.
[Skrive kommentarer, klummer eller artikler.]

- Ikke vigtig
- Lidt vigtig
- Vigtig
- Meget vigtig
- Ekstremt vigtig

326 **4. Task Expertise** [all tasks]

Til hver arbejdsopgave bedes du vurdere din **egen ekspertise** i opgaven.

Ekspertise kan f.eks. komme fra tidligere erfaring med eller naturlige evner for at løse opgaven.

[Skrive kommentarer, klummer eller artikler.]

- Lille ekspertise
- Gennemsnitlig ekspertise
- Stor ekspertise

5. Awareness of ChatGPT

Vi vil nu spørge ind til dine erfaringer med ChatGPT.

Havde du hørt om ChatGPT før denne undersøgelse?

- Ja
- Nej

6. Prior Use of ChatGPT [if 5=’Ja']

Har du benyttet ChatGPT?

- Ja
- Nej

7. Purposes of Prior Use [if 6=’Ja’]

Til hvilke formål har du benyttet ChatGPT?

- Kun arbejde
- Kun fritid
- Både arbejde og fritid

8. Prior Use in Job Tasks [if 7=’Kun arbejde’ or 7=’Både arbejde og fritid’; all tasks]

Har du benyttet ChatGPT til at udføre følgende arbejdsopgaver?

Markér alle opgaver, hvor du har benyttet ChatGPT.

[Skrive kommentarer, klummer eller artikler.]

9. Current Use of ChatGPT [if 6=’Ja’]

Har du benyttet ChatGPT i løbet af de seneste to uger?

- Ja
- Nej

10. Plus Subscription [if 6=’Ja’]

Har du et aktivt Plus-abonnement på ChatGPT?

- Ja
- Nej

327 **Block 2: Prior Beliefs**

11. Prior Beliefs: Productivity Introduction

Tidsbesparelser fra ChatGPT

Vi vil i det følgende spørge til din vurdering af, om ChatGPT kan spare tid i forskellige arbejdsopgaver. Bemærk: Dine svar er vigtige uanset dit kendskab til ChatGPT. Hvis du ikke kender til ChatGPT, beder vi dig give dit bedste gæt. Du vil senere få mulighed for at angive hvor sikker du er i dine vurderinger.

12. Prior Beliefs: Productivity of ChatGPT [all tasks]

Tænk på **en [journalist] med en gennemsnitlig erfaring og ekspertise**, der vil udføre en given arbejdsopgave. Vedkommende har adgang til ChatGPT, internettet, en computer med eksisterende programmer samt andre hjælpemidler, der typisk anvendes i arbejdsopgaven.

Specificér de følgende arbejdsopgaver ud fra beskrivelsen nedenfor. Tilsvarende kvalitet betyder, at hvis andre tjekker arbejdet, vil de ikke kunne vurdere, om opgaven er løst med eller uden hjælp fra ChatGPT.

Stor tidsbesparelse fra ChatGPT

Angiv tidsbesparelse i arbejdsopgaven som ”Stor”, hvis **ChatGPT mindst *kan halvere den tid***, det tager for en gennemsnitlig [journalist] at løse arbejdsopgaven med tilsvarende kvalitet.

Lille eller ingen tidsbesparelse fra ChatGPT

Angiv tidsbesparelse i arbejdsopgaven som ”Lille eller ingen”, hvis ***ChatGPT ikke kan halvere tiden***, det tager for en gennemsnitlig [journalist] at løse arbejdsopgaven med tilsvarende kvalitet.

Angiv venligst dine bedste vurderinger, også selvom du er usikker på dem.

[Skrive kommentarer, klummer eller artikler.]

- Lille eller ingen tidsbesparelse fra ChatGPT
- Stor tidsbesparelse fra ChatGPT

13. Uncertainty of Productivity Prior

Hvor sikker er du i dine forrige vurderinger af tidsbesparelse fra ChatGPT for en gennemsnitlig [journalist]?

- Meget usikker
- Usikker
- Sikker
- Meget sikker

328 **14. Prior Beliefs: Complementarity Introduction**

Vi beder dig nu vurdere, hvordan eventuelle tidsbesparelser fra ChatGPT relaterer sig til [journalisters] ekspertise i de givne arbejdsopgaver.

15. Prior Beliefs: Expertise Complementarity of ChatGPT [all tasks]

Forestil dig to [journalister] med gennemsnitlig erfaring og ekspertise, men som har forskellig ekspertise indenfor én given arbejdsopgave.

En [journalist] med større ekspertise indenfor opgaven: Vedkommende har stor erfaring indenfor netop dén opgave, har indgående kendskab til arbejdsopgavens nuancer og har tidligere løst opgaven effektivitet med stor nøjagtighed.

En [journalist] med mindre ekspertise indenfor arbejdsopgaven: Vedkommende har en bred forståelse for arbejdsopgavens principper, men mangler ekspertise i den konkrete opgave.

De to [journalister] er sammenlignelige i alle andre sammenhænge end deres ekspertise indenfor den specifikke arbejdsopgave.

Specificér følgende arbejdsopgaver ud fra, om brug af ChatGPT kan spare mindre, tilsvarende eller mere tid for medarbejderen med større ekspertise sammenlignet med medarbejderen med mindre ekspertise indenfor opgaven.

Angiv venligst dine bedste vurderinger, også selvom du er usikker på dem.

[Skrive kommentarer, klummer eller artikler.]

- Mindre tidsbesparelse for [journalisten] med større ekspertise
- Samme tidsbesparelse for [journalisten] med større ekspertise
- Større tidsbesparelse for [journalisten] med større ekspertise

16. Uncertainty of Complementarity Prior

Hvor sikker er du i dine forrige vurderinger af, hvordan tidsbesparelse fra ChatGPT relaterer sig til [journalisters] ekspertise?

Meget usikker

Usikker

Sikker

Meget sikker

329 **Block 3: Treatment**

17.a Productivity Treatment [if randomized into productivity treatment group; all tasks]
Vi bad dig tidligere vurdere tidsbesparelsen fra ChatGPT i forskellige arbejdsopgaver.

University of Pennsylvania og OpenAI (udvikleren af ChatGPT) foretog i august 2023 en ekspertvurdering af tidsbesparelsen fra ChatGPT i samme opgaver. Forskere fra Københavns Universitet har valideret og udvidet ekspertvurderingerne i samarbejde med danske brancheeksperter.

Tag dig venligst tid til at gennemgå tabellen, da informationen kan blive nyttig for dig i resten af spørgeskemaet.

Bemærk: Du kan fortsætte ved at klikke på "næste" knappen efter 15 sekunder på denne side. Når du går videre, kan du ikke klikke tilbage til denne tabel.

<table>
<tr><td colspan="2">Skrive kommentarer, klummer eller artikler.</td></tr>
<tr><td colspan="2">Spørgsmål: Kan man spare tid med ChatGPT?</td></tr>
<tr><td>Din vurdering</td><td>Lille eller ingen tidsbesparelse fra ChatGPT</td></tr>
<tr><td>Ekspertvurdering</td><td>Stor tidsbesparelse fra ChatGPT</td></tr>
<tr><td>Forklaring til ekspertvurderingen</td><td>ChatGPT kan generere udkast, foreslå ændringer og give idéer til artikler mv.</td></tr>
<tr><td colspan="2">Spørgsmål: Kan en med større ekspertise spare mere tid?</td></tr>
<tr><td>Din vurdering</td><td>Samme tidsbesparelse for [journalisten] med større ekspertise</td></tr>
</table>

330 **17.b Complementarity Treatment** [if randomized into complementarity treatment group; all tasks]
Vi bad dig tidligere vurdere tidsbesparelsen fra ChatGPT i forskellige arbejdsopgaver.

University of Pennsylvania og OpenAI (udvikleren af ChatGPT) foretog i august 2023 en ekspertvurdering af tidsbesparelsen fra ChatGPT i samme opgaver. Forskere fra Københavns Universitet har valideret og udvidet ekspertvurderingerne i samarbejde med danske brancheeksperter.

Tag dig venligst tid til at gennemgå tabellen, da informationen kan blive nyttig for dig i resten af spørgeskemaet.

Bemærk: Du kan fortsætte ved at klikke på "næste" knappen efter 15 sekunder på denne side. Når du går videre, kan du ikke klikke tilbage til denne tabel.

Skrive kommentarer, klummer eller artikler.	
**Spørgsmål:** Kan man spare tid med ChatGPT?	
Din vurdering	Lille eller ingen tidsbesparelse fra ChatGPT
**Spørgsmål:** Kan en med større ekspertise spare mere tid?	
Din vurdering	Samme tidsbesparelse for [journalisten] med større ekspertise
Ekspertvurdering	Mindre tidsbesparelse for [journalisten] med større ekspertise
Forklaring til **ekspertvurderingen**	En kompetent [journalist] kan nemmere udarbejde udkast til og revidere artikler mv. og har derfor mindre gavn af ChatGPT i opgaven.

331 **17.c Control** [if randomized into control group; all tasks]
Vi bad dig tidligere vurdere tidsbesparelsen fra ChatGPT i forskellige arbejdsopgaver.

Tag dig venligst tid til at gennemgå tabellen, da informationen kan blive nyttig for dig i resten af spørgeskemaet.

Bemærk: Du kan fortsætte ved at klikke på "næste" knappen efter 15 sekunder på denne side. Når du går videre, kan du ikke klikke tilbage til denne tabel.

Skrive kommentarer, klummer eller artikler.	
**Spørgsmål:** Kan man spare tid med ChatGPT?	
Din vurdering	Lille eller ingen tidsbesparelse fra ChatGPT
**Spørgsmål:** Kan en med større ekspertise spare mere tid?	
Din vurdering	Samme tidsbesparelse for [journalisten] med større ekspertise

332 **Block 4: Intended Adoption and Posterior Beliefs**

18. Intentions to Use ChatGPT

Forventer du at benytte ChatGPT i løbet af de næste to uger?

- Ja
- Nej

19. Intentions to Use in Job Tasks [if 18='Ja']

Forventer du at benytte ChatGPT i de følgende arbejdsopgaver de næste to uger?

[Skrive kommentarer, klummer eller artikler.]

- Ja
- Nej

20. Posterior Beliefs: Individual Productivity of ChatGPT [all tasks]

Tidsbesparelse fra ChatGPT i eget job

Tag nu udgangspunkt i **dit eget job**, givet din egen erfaring og ekspertise som [journalist]. Antag, at du har adgang til ChatGPT, internettet, en computer med eksisterende programmer, samt andre hjælpemidler, du typisk anvender til at udføre en given arbejdsopgave.

Specificér de følgende arbejdsopgaver ud fra beskrivelsen nedenfor. Tilsvarende kvalitet betyder, at hvis andre tjekker arbejdet vil de ikke kunne vurdere, om du har løst opgaven med eller uden hjælp fra ChatGPT.

Stor tidsbesparelse fra ChatGPT

Angiv tidsbesparelse i arbejdsopgaven som "Stor", hvis ***ChatGPT mindst kan halvere tiden***, det tager for dig at løse arbejdsopgaven med tilsvarende kvalitet.

Lille eller ingen tidsbesparelse fra ChatGPT

Angiv tidsbesparelse i arbejdsopgaven som "Lille eller ingen", hvis ***ChatGPT ikke kan halvere tiden***, det tager for dig at løse arbejdsopgaven med tilsvarende kvalitet.

Angiv venligst dine bedste vurderinger, også selvom du er usikker på dem.

[Skrive kommentarer, klummer eller artikler.]

- Lille eller ingen tidsbesparelse fra ChatGPT
- Stor tidsbesparelse fra ChatGPT

333 **21. Uncertainty of Individual Productivity Posterior**
Hvor sikker er du i dine forrige vurderinger af tidsbesparelsen fra ChatGPT for dig selv?

- Meget usikker
- Usikker
- Sikker
- Meget sikker

22. Final Questions: Introduction
Vi vil her til sidst stille dig nogle opfølgende spørgsmål om din vurdering af ChatGPT i de forskellige arbejdsopgaver.

23.a Task Substitution
Hvis ChatGPT sparer tid i løsningen af en opgave, forventer du så at løse flere af den type opgaver i løbet af din arbejdsdag?
Angiv venligst din bedste vurdering, også selvom du er usikker på den.

- Ja
- Nej

23.b Task Substitution [if 23.a='Ja']
Hvis ChatGPT sparer tid i løsningen af en opgave, forventer du så, at den type opgaver vil fylde mere i din arbejdsdag, mens andre slags opgaver vil fylde mindre?
Opgaven kan fylde mere, hvis det større antal løste opgaver opvejer tidsbesparelsen i den enkelte opgaveløsning.

- Ja
- Nej

Block 5: Frictions

24. Beliefs Frictions [tasks with 12!=20]
Din vurdering af tidsbesparelserne fra ChatGPT for en gennemsnitlig [journalist] og dig selv var forskellige fra hinanden i de følgende arbejdsopgaver.

Angiv til hver af opgaverne årsagerne til, at dine vurderinger adskilte sig fra hinanden.

[Skrive kommentarer, klummer eller artikler.]

- Jeg ændrede mit syn på tidsbesparelsen fra ChatGPT.
- Tidsbesparelsen for en gennemsnitlig [journalist] er ikke relevant givet min ekspertise.
- Jeg tror ikke, at jeg kan finde ud af at bruge ChatGPT.
- Jeg anvender specialiseret software, der ikke kan integreres med ChatGPT.
- Jeg er bekymret for, om ChatGPTs svar er korrekte.
- Jeg er bekymret for ChatGPT's manglende evner på dansk.
- Andet, skriv venligst: [open text field]

334 **S25: Adoption Frictions** [tasks with 19=’Nej’ and 20=’Stor tidsbesparelse fra ChatGPT’]
Du angav for følgende arbejdsopgaver, at
1. ChatGPT kan give dig store tidsbesparelser i opgaveløsningen.
2. Du ikke forventer at benytte ChatGPT i opgaven de næste to uger.

Angiv årsager til, at du ikke forventer at benytte ChatGPT i arbejdsopgaven på trods af dens tidsbesparelser

[Skrive kommentarer, klummer eller artikler.]

- Jeg forventer ikke at stå over for opgaven de næste to uger.
- Jeg er pålagt restriktioner om brugen af ChatGPT i mit job.
- Jeg er bekymret for, om ChatGPT varetager mine data fortroligt.
- Det vil kræve oplæring, før jeg kan få gavn af ChatGPT.
- Jeg frygter, at ChatGPT på sigt vil gøre mig overflødig i jobbet.
- ChatGPT vil mindske min fornøjelse ved at udføre opgaven
- Jeg er bekymret for at blive afhængig af ChatGPT i opgaveløsningen
- Andet, skriv venligst: [open text field]

26. Information Sheets
Er du interesseret i at modtage uddybende materiale med eksempler på, hvordan en [journalist] kan anvende ChatGPT?
Materialet er udarbejdet af forskere fra Københavns Universitet og University of Chicago i samarbejde med danske brancheeksperter. Du kan tilgå materialet i slutningen af spørgeskemaet

- Ja
- Nej

Side 27.(a) End of Survey [if 26=’Ja’]
Mange tak for at deltage i undersøgelsen.
Du kan hente materialet, der er udarbejdet af forskere fra Københavns Universitet og University of Chicago i samarbejde med danske brancheeksperter, her: **ChatGPT for [journalister]**
Hvis du vinder en af præmierne, vil du få direkte besked i din e-Boks.
Vi vil muligvis kontakte dig igen om to uger med en kort opfølgningsundersøgelse. Vi håber meget, at du er villig til at deltage i denne korte opfølgning.

Side 27.(b) End of Survey [if 26=’Nej’]
Mange tak for at deltage i undersøgelsen.
Hvis du vinder en af præmierne, vil du få direkte besked i din e-Boks.
Vi vil muligvis kontakte dig igen om to uger med en kort opfølgningsundersøgelse. Vi håber meget, at du er villig til at deltage i denne korte opfølgning.

9. Expert Assessments

This section shows the expert assessments and explanations for each job task in our survey. Table S24 provides the English translations and Table S25 is the original Danish versions.

Table S24. Expert Assessments of Time Savings from ChatGPT (English Translation)

Occupation	Job Task	Time Savings	Explanation
Accountants & Auditors	Prepare detailed reports on audit findings.	Large	ChatGPT can draft and structure reports based on audit findings.
Accountants & Auditors	Supervise auditing of establishments, and determine scope of investigation required.	Small or no	The task requires human interaction and decision-making, which is beyond ChatGPT's capabilities.
Accountants & Auditors	Examine and evaluate financial and information systems, recommending controls to ensure system reliability and data integrity.	Large	ChatGPT can compile reports on financial and IT systems from provided data and text, identifying potential issues and suggesting controls for system reliability and data integrity.
Accountants & Auditors	Confer with company officials about financial and regulatory matters.	Small or no	The task requires human interaction, which is beyond ChatGPT's capabilities.
Accountants & Auditors	Prepare, examine, or analyze accounting records, financial statements, or other financial reports to assess accuracy, completeness, and conformance to reporting and procedural standards.	Large	ChatGPT can draft accounting documents and analyze accounting information and financial reports.
Accountants & Auditors	Compute taxes owed and prepare tax returns, ensuring compliance with payment, reporting, or other tax requirements.	Large	ChatGPT can provide guidance on tax legislation, calculate tax liabilities, and generate drafts of tax returns.
Customer Service Rep.	Confer with customers by telephone or in person to provide information about products or services, take or enter orders, cancel accounts, or obtain details of complaints.	Small or no	ChatGPT cannot speak with customers in person or over the phone.
Customer Service Rep.	Keep records of customer interactions or transactions, recording details of inquiries, complaints, or comments, as well as actions taken.	Large	ChatGPT can assist with logging and reporting customer contacts based on data from customer support.
Customer Service Rep.	Check to ensure that appropriate changes were made to resolve customers' problems.	Large	ChatGPT can prepare a structured report on whether the actions taken resolved the customer complaint.
Customer Service Rep.	Contact customers to respond to inquiries or to notify them of claim investigation results or any planned adjustments.	Large	ChatGPT can suggest responses to customer inquiries and complaints.
Customer Service Rep.	Determine charges for services requested, collect deposits or payments, or arrange for billing.	Small or no	ChatGPT cannot collect payments or deposits without additional software, but it can help set prices and issue invoices.
Customer Service Rep.	Review claims adjustments with dealers, examining parts claimed to be defective, and approving or disapproving dealers' claims.	Small or no	ChatGPT cannot inspect physical products or make final decisions regarding dealers.
Financial Advisors	Interview clients to determine their current income, expenses, insurance coverage, tax status, financial objectives, risk tolerance, or other information needed to develop a financial plan.	Small or no	The task requires human interaction, which is beyond ChatGPT's capabilities.
Financial Advisors	Recommend to clients strategies in cash management, insurance coverage, investment planning, or other areas to help them achieve their financial goals.	Large	ChatGPT can develop and formulate financial strategies and plans based on a client's financial situation and goals.
Financial Advisors	Manage client portfolios, keeping client plans up-to-date.	Small or no	ChatGPT cannot load financial data in real-time or make portfolio management decisions. However, it can generate reports on client portfolios and suggest strategies for portfolio management.
Financial Advisors	Implement financial planning recommendations, or refer clients to someone who can assist them with plan implementation.	Large	ChatGPT can provide step-by-step instructions for implementing a client's financial plan and suggest agents who can assist with the implementation.
Financial Advisors	Analyze financial information obtained from clients to determine strategies for meeting clients' financial objectives.	Large	ChatGPT can suggest and describe suitable financial strategies based on clients' financial situations and goals.
Financial Advisors	Answer clients' questions about the purposes and details of financial plans and strategies.	Large	ChatGPT can suggest answers to typical questions about financial plans and strategies.

Table S24 (Continued): Expert Assessments of Time Savings from ChatGPT (English Translation)

Occupation	Job Task	Time Savings	Explanation
HR Professionals	Interpret and explain human resources policies, procedures, laws, standards, or regulations.	Large	ChatGPT can interpret and explain complex HR policies and regulations in easily accessible language.
HR Professionals	Hire employees and process hiring-related paperwork.	Small or no	The task requires human interaction and decision-making, which is beyond ChatGPT's capabilities, but it can automate employment-related paperwork.
HR Professionals	Inform job applicants of details such as duties and responsibilities, compensation, benefits, schedules, working conditions, or promotion opportunities.	Large	ChatGPT can generate detailed descriptions of jobs and employment terms.
HR Professionals	Prepare or maintain employment records related to events, such as hiring, termination, leaves, transfers, or promotions, using human resources management system software.	Small or no	ChatGPT cannot interact with HR software, but it can assist with text descriptions in contracts and similar documents.
HR Professionals	Address employee relations issues, such as harassment allegations, work complaints, or other employee concerns.	Small or no	The task requires human interaction and decision-making, which is beyond ChatGPT's capabilities.
HR Professionals	Schedule or conduct new employee orientations.	Small or no	The task requires human interaction and decision-making, which is beyond ChatGPT's capabilities.
IT Support	Answer user inquiries regarding computer software or hardware operation to resolve problems.	Large	ChatGPT can provide step-by-step instructions for solving typical hardware and software problems.
IT Support	Oversee the daily performance of computer systems.	Small or no	The task requires real-time monitoring and decision-making, which is beyond ChatGPT's capabilities, but it can analyze log files.
IT Support	Read technical manuals, confer with users, or conduct computer diagnostics to investigate and resolve problems or to provide technical assistance and support.	Large	ChatGPT can summarize technical manuals and assist with technical support by suggesting questions to users and possible solutions.
IT Support	Set up equipment for employee use, performing or ensuring proper installation of cables, operating systems, or appropriate software.	Small or no	The task requires physical work, which is beyond ChatGPT's capabilities, but it can provide instructions for the work.
IT Support	Enter commands and observe system functioning to verify correct operations and detect errors.	Small or no	ChatGPT cannot monitor or interact with other computer systems in real-time.
IT Support	Maintain records of daily data communication transactions, problems and remedial actions taken, or installation activities.	Large	ChatGPT can structure log files and notes into coherent reports.
Journalists	Write commentaries, columns, or scripts.	Large	ChatGPT can generate drafts, suggest changes, and provide ideas for articles, etc.
Journalists	Coordinate and serve as an anchor on news broadcast programs.	Small or no	The task requires human interaction, presence, and decision-making, which is beyond ChatGPT's capabilities.
Journalists	Examine news items of local, national, and international significance to determine topics to address, or obtain assignments from editorial staff members.	Large	ChatGPT can analyze and summarize news content and suggest topics to cover.
Journalists	Analyze and interpret news and information received from various sources to broadcast the information.	Large	ChatGPT can analyze, summarize, and translate news from various sources.
Journalists	Arrange interviews with people who can provide information about a story.	Small or no	ChatGPT cannot interact with people or manage calendars, but it can draft interview invitations.
Journalists	Present news stories, and introduce in-depth videotaped segments or live transmissions from on-the-scene reporters.	Small or no	The task requires physical presence, which is beyond ChatGPT's capabilities.
Legal Professionals	Prepare affidavits or other documents, such as legal correspondence, and organize and maintain documents in paper or electronic filing system.	Large	ChatGPT can suggest templates and drafts for legal documents and provide guidance on filing.
Legal Professionals	Prepare legal documents, including briefs, pleadings, appeals, wills, contracts, and real estate closing statements.	Large	ChatGPT can deliver drafts of legal documents based on entered details.
Legal Professionals	Prepare for trial by performing tasks such as organizing exhibits.	Small or no	The task requires human interaction, physical work, and decision-making, which is beyond ChatGPT's capabilities.
Legal Professionals	Investigate facts and law of cases and search pertinent sources, such as public records and internet sources, to determine causes of action and to prepare cases.	Small or no	ChatGPT cannot interact with databases, registries, or the internet without additional software.
Legal Professionals	Meet with clients and other professionals to discuss details of case.	Small or no	The task requires physical presence and human interaction, which is beyond ChatGPT's capabilities.
Legal Professionals	File pleadings with court clerk.	Small or no	The task requires the submission of physical documents or interaction with other online systems, which is beyond ChatGPT's capabilities.

Table S24 (Continued): Expert Assessments of Time Savings from ChatGPT (English Translation)

Occupation	Job Task	Time Savings	Explanation
Marketing Professionals	Prepare reports of findings, illustrating data graphically and translating complex findings into written text.	Large	ChatGPT can write and structure reports from data and text, and can also suggest presentation forms for data.
Marketing Professionals	Collect and analyze data on customer demographics, preferences, needs, and buying habits to identify potential markets and factors affecting product demand.	Small or no	ChatGPT cannot collect data or interact with databases, but it can summarize and analyze text describing data.
Marketing Professionals	Conduct research on consumer opinions and marketing strategies, collaborating with marketing professionals, statisticians, pollsters, and other professionals.	Small or no	ChatGPT cannot conduct surveys or interact directly with people, but it can suggest survey questions and draft reports.
Marketing Professionals	Measure and assess customer and employee satisfaction.	Small or no	ChatGPT cannot conduct surveys or collect data, but it can suggest questionnaires and generate drafts of summary reports for the survey.
Marketing Professionals	Measure the effectiveness of marketing, advertising, and communications programs and strategies.	Small or no	ChatGPT cannot measure the effectiveness of marketing strategies without additional software, but it can suggest strategies for the study and generate draft reports.
Marketing Professionals	Attend staff conferences to provide management with information and proposals concerning the promotion, distribution, design, and pricing of company products or services.	Small or no	The task requires human interaction, which is beyond ChatGPT's capabilities.
Office Clerks	Operate office machines, such as computers, voice mail systems, photocopiers, and scanners.	Small or no	The task requires physical work, which is beyond ChatGPT's capabilities.
Office Clerks	Answer telephones, direct calls, and take messages.	Small or no	ChatGPT cannot operate telephones.
Office Clerks	Communicate with customers, employees, and other individuals to answer questions, disseminate or explain information, take orders, and address complaints.	Large	ChatGPT can suggest responses to typical inquiries, complaints, and orders.
Office Clerks	Compile, copy, sort, and file records of office activities, business transactions, and other activities.	Large	ChatGPT can prepare records following complex instructions and assist with filing and sorting documents by summarizing and editing text.
Office Clerks	Open, sort, and route incoming mail, answer correspondence, and prepare outgoing mail.	Small or no	The task requires physical work, which is beyond ChatGPT's capabilities.
Office Clerks	Compute, record, and proofread data and other information, such as records or reports.	Large	ChatGPT can prepare and check records and reports based on predefined guidelines.
Software Developers	Write, analyze, review, and rewrite programs, using workflow chart and diagram, and applying knowledge of computer capabilities, subject matter, and symbolic logic.	Large	ChatGPT can assist with writing code and analyzing errors in programs based on software developers' preferences and program outputs.
Software Developers	Correct errors by making appropriate changes and rechecking the program to ensure that the desired results are produced.	Large	ChatGPT can identify code errors and suggest corrections and checks based on error messages and other program outputs.
Software Developers	Perform or direct revision, repair, or expansion of existing programs to increase operating efficiency or adapt to new requirements.	Large	ChatGPT can provide code suggestions for auditing, debugging, and extending programs, and can also suggest ways to optimize the code.
Software Developers	Consult with managerial, engineering, and technical personnel to clarify program intent, identify problems, and suggest changes.	Small or no	The task requires human interaction, which is beyond ChatGPT's capabilities.
Software Developers	Conduct trial runs of programs and software applications to be sure they will produce the desired information and that the instructions are correct.	Large	ChatGPT can suggest code changes and debug programs, as well as explain program output in a reader-friendly format.
Software Developers	Consult with and assist computer operators or system analysts to define and resolve problems in running computer programs.	Large	ChatGPT can identify code errors and suggest corrections based on error messages, program output, and user input.
Teachers	Prepare students for later grades by encouraging them to explore learning opportunities and to persevere with challenging tasks.	Small or no	The task requires human interaction and understanding of the student's needs, which is beyond ChatGPT's capabilities.
Teachers	Adapt teaching methods and instructional materials to meet students' varying needs and interests.	Large	ChatGPT can tailor teaching methods and materials based on each student's learning style and interests.
Teachers	Establish and enforce rules for behavior and procedures for maintaining order among students.	Small or no	The task requires human interaction and understanding of students' behavior, which is beyond ChatGPT's capabilities.
Teachers	Prepare objectives and outlines for courses of study, following curriculum guidelines or requirements of states and schools.	Large	ChatGPT can suggest and structure learning objectives and courses in accordance with curricula or similar requirements.
Teachers	Prepare, administer, and grade tests and assignments to evaluate students' progress.	Small or no	Administering exams requires physical work, which is beyond ChatGPT's capabilities. ChatGPT can help formulate and assess exams and assignments, but automating many assessments would require additional software.
Teachers	Prepare materials and classrooms for class activities.	Small or no	The task requires physical work, which is beyond ChatGPT's capabilities.

338

Table S25. Expert Assessments of Time Savings from ChatGPT (Original Danish Version)

Faggruppe	Opgave	Tidsbesparelse	Forklaring
Revisions- og regnskabsmedarbejder	Udarbejde detaljerede revisionsrapporter.	Stor	ChatGPT kan levere udkast til og strukturere rapporter baseret på revisionsresultater.
Revisions- og regnskabsmedarbejder	Overvåge revision af virksomheder og fastsætte omfanget af en sådan undersøgelse.	Lille eller ingen	Opgaven kræver menneskelig interaktion og beslutningstagning, hvilket er uden for ChatGPT's evner.
Revisions- og regnskabsmedarbejder	Undersøge finansielle systemer eller IT-systemer og anbefale kontroller af systemers pålidighed og dataintegritet.	Stor	ChatGPT kan udarbejde rapporter om finansieller og IT-systemer baseret på inputdata og -tekst. ChatGPT kan derudfra identificere potentielle problemer med og foreslå kontroller af systemers pålidighed og dataintegritet.
Revisions- og regnskabsmedarbejder	Rådføre sig med virksomhedsledelsen om økonomiske og regulatoriske forhold.	Lille eller ingen	Opgaven kræver menneskelig interaktion, hvilket er uden for ChatGPT's evner.
Revisions- og regnskabsmedarbejder	Forberede, undersøge eller analysere regnskabsdokumenter eller andre finansielle rapporter for at vurdere nøjagtighed, fuldstændighed samt overholdelse af rapporterings- og proceduremæssige standarder.	Stor	ChatGPT kan udarbejde udkast til regnskabsdokumenter samt analysere regnskabsoplysninger og finansielle rapporter.
Revisions- og regnskabsmedarbejder	Beregne skatteforpligtelser og udarbejde selvangivelser i overensstemmelse med skatteregler.	Stor	ChatGPT kan vejlede om skattelovgivning, beregne skatteforpligtelser og generere udkast til selvangivelser.
Kundesupporter	Tale med kunder personligt eller telefonisk for at informere om produkter eller tjenester, modtage ordrer, opsige konti eller indhente detaljer om kundeklager.	Lille eller ingen	ChatGPT kan ikke tale med kunder personligt eller telefonisk.
Kundesupporter	Registrere kontakt eller transaktioner med kunder vedrørende detaljer om forespørgsler, klager eller kommentarer samt besluttede foranstaltninger.	Stor	ChatGPT kan hjælpe med registrering og afrapportering af kundekontakt baseret på data fra kundesupporteren.
Kundesupporter	Kontrollere at passende foranstaltninger blev foretaget for at løse kunders problemer.	Stor	ChatGPT kan sammenholde aftalte foranstaltningerne med kundeklagen samt udarbejde en struktureret rapport for om foranstaltningerne løste kundens problemer.
Kundesupporter	Kontakte kunder for at besvare forespørgsler eller informere dem om udfaldet af klageundersøgelser eller planlagte foranstaltninger.	Stor	ChatGPT kan foreslå svar på kundeforespørgsler og klagesager.
Kundesupporter	Fastlægge pris på efterspurgte ydelser, udstede fakturaer, eller indsamle deposita og betalinger.	Lille eller ingen	ChatGPT kan ikke indsamle betalinger eller deposita uden yderligere software. ChatGPT kan dog fastsætte priser samt udstede fakturaer.
Kundesupporter	Gennemgå reklamationer med forhandlere, undersøge hævdede fejl i produkter, og godkende eller afvise forhandleres krav.	Lille eller ingen	ChatGPT kan ikke inspicere fysiske produkter eller tage endelige beslutninger over for forhandlere.
Økonomisk rådgiver	Interviewe klienter for at afgøre deres nuværende indkomst, udgifter, forsikringsdækning, skattestatus, økonomiske mål, risikotolerance eller andre oplysninger, der er nødvendige for at udvikle en økonomisk plan.	Lille eller ingen	Opgaven kræver menneskelig interaktion, hvilket er uden for ChatGPT's evner.
Økonomisk rådgiver	Anbefale klienter strategier inden for likviditetsstyring, forsikringsdækning, investeringsplanlægning eller andre områder for at hjælpe dem med at opnå deres økonomiske mål.	Stor	ChatGPT kan udvikle og formulere finansielle strategier og planer baseret på klientens økonomiske situation og mål.
Økonomisk rådgiver	Forvalte klientporteføljer og holde klientplaner opdaterede.	Lille eller ingen	ChatGPT kan ikke indlæse finansielle data i realtid eller træffe beslutninger om porteføljeforvaltning. ChatGPT kan dog generere rapporter om klientporteføljer samt foreslå strategier til porteføljeforvaltningen.
Økonomisk rådgiver	Gennemføre anbefalinger fra en økonomisk plan eller henvise klienter til nogen, der kan hjælpe dem med implementeringen.	Stor	ChatGPT kan give trinvise instruktioner, der implementerer en klients økonomiske plan. ChatGPT kan desuden foreslå aktører, der kan hjælpe med implementeringen.
Økonomisk rådgiver	Analysere økonomiske oplysninger om klienter for at fastlægge strategier, der opfylder klienters økonomiske mål.	Stor	ChatGPT kan foreslå og beskrive passende økonomiske strategier baseret på kunders finansielle situation og mål.
Økonomisk rådgiver	Besvare klienters spørgsmål om formålet for og detaljerne i økonomiske planer og strategier.	Stor	ChatGPT kan foreslå svar på typiske spørgsmål om økonomiske planer og strategier.

Table S25 (Continued): Expert Assessments of Time Savings from ChatGPT (Original Danish Version)

Faggruppe	Opgave	Tidsbesparelse	Forklaring
HR-medarbejder	Fortolke og forklare politikker, procedurer, love, standarder eller reguleringer inden for HR-området.	Stor	ChatGPT kan fortolke og forklare komplekse HR-politikker og -reguleringer i et let tilgængeligt sprog.
HR-medarbejder	Ansætte medarbejdere og behandle ansættelsesrelateret papirarbejde.	Lille eller ingen	Opgaven kræver menneskelig interaktion og beslutningstagen, hvilket er uden for ChatGPT's evner. ChatGPT kan dog automatisere ansættelsesrelateret papirarbejde.
HR-medarbejder	Informere jobansøgere om ansættelsesvilkår, såsom jobindhold og -ansvar, løn og goder, arbejdstider og -forhold samt muligheder for forfremmelse.	Stor	ChatGPT kan generere detaljerede beskrivelser om jobbet og ansættelsesvilkår.
HR-medarbejder	Opbygge eller vedligeholde ansættelsesregistre i forbindelse med ansættelse, fratrædelse, orlov, omplacering eller forfremmelser ved hjælp af HR-software.	Lille eller ingen	ChatGPT kan ikke interagere med HR-software. ChatGPT kan dog hjælpe med tekstbeskrivelser i kontrakter mv.
HR-medarbejder	Håndtere medarbejderrelationer, herunder sager om chikane, arbejdsrelaterede klager eller andre bekymringer fra medarbejdere.	Lille eller ingen	Opgaven kræver menneskelig interaktion og beslutningstagning, hvilket er uden for ChatGPT's evner.
HR-medarbejder	Planlægge eller gennemføre introduktioner for nye medarbejdere.	Lille eller ingen	Opgaven kræver menneskelig interaktion og beslutningstagning, hvilket er uden for ChatGPT's evner.
IT-supporter	Besvare brugerhenvendelser vedrørende drift af computerhardware eller -software for at løse problemer.	Stor	ChatGPT kan give trinvise instruktioner til løsninger på typiske hardware- og softwareproblemer.
IT-supporter	Overvåge daglig performance af computersystemer.	Lille eller ingen	Opgaven kræver realtidsmonitorering og beslutningstagning, hvilket er uden for ChatGPT's evner. ChatGPT kan dog analysere logfiler.
IT-supporter	Læse tekniske manualer, kommunikere med brugere eller udføre computerdiagnostik for at undersøge og løse problemer eller yde teknisk hjælp og support.	Stor	ChatGPT kan opsummere tekniske manualer samt hjælpe med teknisk support ved at foreslå spørgsmål til brugeren og mulige løsninger.
IT-supporter	Opsætte udstyr til medarbejdere og udføre eller sikre korrekt installation af kabler, operativsystemer eller software.	Lille eller ingen	Opgaven kræver fysisk arbejde, hvilket er uden for ChatGPT's evner. ChatGPT kan dog give instruktioner til arbejdet.
IT-supporter	Indtaste kommandoer og observere computersystemers funktion for at bekræfte korrekt drift eller registrere fejl.	Lille eller ingen	ChatGPT kan ikke monitorere eller interagere med andre computersystemer i realtid.
IT-supporter	Føre log over daglige dataoverførsler, installationer og tekniske problemer samt afhjælpende foranstaltninger.	Stor	ChatGPT kan strukturere logfiler og noter til sammenhængende rapporter.
Journalist	Skrive kommentarer, klummer eller artikler.	Stor	ChatGPT kan generere udkast, foreslå ændringer og give idéer til artikler mv.
Journalist	Være vært på og tilrettelægge nyhedsudsendelser.	Lille eller ingen	Opgaven kræver menneskelig interaktion, tilstedeværelse og beslutningstagning, hvilket er uden for ChatGPT's evner.
Journalist	Gennemgå nyheder af lokal, national og international betydning for at fastlægge hvilke emner, der skal behandles eller få tildelt historier fra redaktionen.	Stor	ChatGPT kan analysere og opsummere nyhedsindhold samt foreslå emner til behandling.
Journalist	Analysere og fortolke nyheder og information fra forskellige kilder for at videreformidle informationen.	Stor	ChatGPT kan analysere, opsummere og oversætte nyheder fra forskellige kilder.
Journalist	Arrangere interviews med personer, der kan give information om en historie.	Lille eller ingen	ChatGPT kan ikke interagere med mennesker eller administrere kalendere. ChatGPT kan dog formulere interviewinvitationer.
Journalist	Præsentere nyhedshistorier og introducere dybdegående videosegmenter eller live transmissioner fra journalister på stedet.	Lille eller ingen	Opgaven kræver fysisk tilstedeværelse, hvilket er uden for ChatGPT's evner.
Juridisk medarbejder	Forberede erklæringer eller andre dokumenter såsom juridisk korrespondance, organisere og vedligeholde dokumenter i et papirbaseret eller elektronisk arkivsystem.	Stor	ChatGPT kan foreslå skabeloner og udkast til juridiske dokumenter samt give vejledning til arkivering.
Juridisk medarbejder	Forberede juridiske dokumenter, herunder processkrifter, forsvars- og anklageskrifter, appeller, testamenter, kontrakter og ejendomshandelsdokumenter.	Stor	ChatGPT kan levere udkast til juridiske dokumenter baseret på indtastede detaljer.
Juridisk medarbejder	Forberede en retssag såsom at udarbejde bevismateriale.	Lille eller ingen	Opgaven kræver menneskelig interaktion, fysisk arbejde og beslutningstagning, hvilket er uden for ChatGPT's evner.
Juridisk medarbejder	Undersøge fakta, omstændigheder og lovgivning i sager og søge relevante kilder, såsom offentlige registre og internetkilder, for at afklare årsager til søgsmål og forberede sager.	Lille eller ingen	ChatGPT kan ikke interagere med databaser, registre eller internettet uden yderligere software.
Juridisk medarbejder	Møde klienter og fagfolk for at drøfte sagsdetaljer.	Lille eller ingen	Opgaven kræver fysisk tilstedeværelse og menneskelig interaktion, hvilket er uden for ChatGPT's evner.
Juridisk medarbejder	Indsende processkrifter til retssekretæren.	Lille eller ingen	Opgaven kræver indsendelse af fysiske dokumenter eller interaktion med andre online systemer, hvilket er uden for ChatGPT's evner.

Table S25 (Continued): Expert Assessments of Time Savings from ChatGPT (Original Danish Version)

Faggruppe	Opgave	Tidsbesparelse	Forklaring
Marketing medarbejder	Udarbejde rapporter, der illustrerer data grafisk og formidler komplekse sammenhænge i tekst.	Stor	ChatGPT kan skrive og strukturere rapporter ud fra data og tekst. ChatGPT kan tilmed foreslå præsentationsformer for data.
Marketing medarbejder	Indsamle og analysere data om kundeoplysninger, præferencer, behov og købsvaner for at identificere potentielle markeder og faktorer, der påvirker produkters efterspørgsel.	Lille eller ingen	ChatGPT kan ikke indsamle data eller interagere med databaser. ChatGPT kan dog opsummere og analysere tekst, der beskriver data.
Marketing medarbejder	Undersøge forbrugerholdninger og marketingstrategier i samarbejde med marketingmedarbejdere, statistikere, meningsmålere og andre fagfolk.	Lille eller ingen	ChatGPT kan ikke udføre undersøgelser eller interagere direkte med mennesker. ChatGPT kan dog foreslå undersøgelsesspørgsmål og udarbejde rapporter.
Marketing medarbejder	Måle og vurdere kunde- og medarbejdertilfredshed.	Lille eller ingen	ChatGPT kan ikke udføre undersøgelser eller indsamle data. ChatGPT kan dog foreslå spørgeskemaer samt generere udkast til opsummerende rapporter for undersøgelsen.
Marketing medarbejder	Måle effekten af marketing, reklame og kommunikationsindsatser og -strategier.	Lille eller ingen	ChatGPT kan ikke måle effekten af marketingstrategier mv. uden yderligere software. ChatGPT kan dog foreslå strategier for undersøgelsen samt generere udkast til rapporter.
Marketing medarbejder	Deltage i medarbejdermøder for at give information og forslag vedrørende markedsføring, distribution, design og prissætning af virksomhedens produkter eller tjenester.	Lille eller ingen	Opgaven kræver menneskelig interaktion, hvilket er uden for ChatGPT's evner.
Kontoransat eller sekretær	Betjene kontormaskiner såsom computere, telefonsvarere, kopimaskiner og scannere.	Lille eller ingen	Opgaven kræver fysisk arbejde, hvilket er uden for ChatGPT's evner.
Kontoransat eller sekretær	Besvare telefonopkald, viderestille opkald og modtage beskeder.	Lille eller ingen	ChatGPT kan ikke betjene telefoner.
Kontoransat eller sekretær	Kommunikere med kunder, brugere, medarbejdere og andre for at besvare spørgsmål, formidle eller forklare information, modtage ordrer og håndtere klager.	Stor	ChatGPT kan foreslå svar på typiske forespørgsler, klager og ordrer.
Kontoransat eller sekretær	Udarbejde, sortere og arkivere optegnelser over kontor-, forretnings- og andre aktiviteter.	Stor	ChatGPT kan udarbejde optegnelser efter komplekse instruktioner samt assistere arkivering og sortering af dokumenterne ved at opsummere og redigere tekst.
Kontoransat eller sekretær	Åbne, sortere og videresende indgående post, besvare henvendelser og forberede udgående post.	Lille eller ingen	Opgaven kræver fysisk arbejde, hvilket er uden for ChatGPT's evner.
Kontoransat eller sekretær	Bearbejde, registrere og tjekke data og anden information såsom optegnelser eller rapporter.	Stor	ChatGPT kan udarbejde og tjekke optegnelser og rapporter baseret på foruddefinerede retningslinjer.
Softwareudvikler	Skrive, analysere, gennemgå og ændre programmer, fx. ved hjælp af rutediagrammer samt ved anvendelse af viden om emnet, computers kapacitet og symbolsk logik.	Stor	ChatGPT kan hjælpe med at skrive kode og analysere fejl i programmer baseret på softwareudviklerens ønsker og programoutput.
Softwareudvikler	Rette fejl i programmer og kontrollere, at ønskede resultater opnås.	Stor	ChatGPT kan identificere kodefejl samt foreslå rettelser og kontroller ud fra fejlmeddelelser og andet programoutput.
Softwareudvikler	Udføre eller lede revision, fejlretning eller udvidelse af eksisterende programmer for at øge driftseffektiviteten eller møde nye krav.	Stor	ChatGPT kan give kodeforslag til revision, fejlretning og udvidelse af programmer. ChatGPT kan desuden foreslå måder at optimere koden.
Softwareudvikler	Konsultere ledelses-, ingeniør- og teknisk personale for at afklare målet for et computerprogram, identificere problemer og foreslå ændringer.	Lille eller ingen	Arbejdsopgaven kræver menneskelig interaktion, hvilket er uden for ChatGPT's evner.
Softwareudvikler	Teste programmer og softwareapplikationer for at sikre, at de genererer det ønskede output og at instruktionerne er korrekte.	Stor	ChatGPT kan foreslå kodeændringer og debugge programmer samt forklare programoutput i et læsevenligt format.
Softwareudvikler	Konsultere og hjælpe IT-teknikere eller systemanalytikere med at identificere og løse problemer i forbindelse med kørsel af computerprogrammer.	Stor	ChatGPT kan identificere kodefejl samt foreslå rettelser ud fra fejlmeddelelser, programoutput samt input fra brugeren.
Lærer	Forberede eleverne til senere klassetrin ved at motivere dem til at udforske læringsmuligheder og støtte dem i at løse udfordrende opgaver.	Lille eller ingen	Opgaven kræver menneskelig interaktion samt forståelse for elevens behov, hvilket er uden for ChatGPT's evner.
Lærer	Tilpasse undervisningsmetoder og -materialer for at imødekomme elevers forskellige behov og interesser.	Stor	ChatGPT kan målrette undervisningsmetoder og -materialer baseret på den enkelte elevs læringsstil og interesser.
Lærer	Fastlægge og håndhæve regler for adfærd og procedurer for at opretholde orden blandt eleverne.	Lille eller ingen	Opgaven kræver menneskelig interaktion samt forståelse for elevers adfærd, hvilket er uden for ChatGPT's evner.
Lærer	Forberede undervisningsmål og -forløb i overensstemmelse med læseplaner eller krav fra stat, kommune eller skole.	Stor	ChatGPT kan foreslå og strukturere læringsmål og -forløb i overensstemmelse med læseplaner eller lignende krav.
Lærer	Formulere, afholde og bedømme prøver og opgaver for at vurdere elevers udvikling.	Lille eller ingen	Afholdelse af prøver kræver fysisk arbejde, hvilket er uden for ChatGPT's evner. ChatGPT kan hjælpe med at formulere og bedømme prøver og opgaver, men automatisering af mange bedømmelser vil kræve yderligere software.
Lærer	Indrette klasseværelser og fysiske materialer til undervisningsaktiviteter.	Lille eller ingen	Opgaven kræver fysisk arbejde, hvilket er uden for ChatGPT's evner.

10. Information Sheets

We create information sheets for each of the 11 occupations, containing three ChatGPT prompts that exemplify use cases in their exposed job tasks. We use GPT to generate example prompts for the exposed job tasks, which we then manually reviewed and tested. In the survey, workers may sign up for the sheets, thus revealing their interest in information about using ChatGPT. We also track participants' eventual clicks on the hyperlinked materials. The following page shows the information sheet for journalists (original Danish version). The remaining information sheets are available at www.andershumlum.com/s/sheets.zip.

346

KØBENHAVNS
UNIVERSITET

ChatGPT for journalister

ChatGPT er en chatbot med kunstig intelligens. Arket her hjælper dig i gang med at bruge værktøjet.

Tilgå ChatGPT

1. Besøg **chat.openai.com**.
2. Følg instruktionerne på skærmen for at oprette en gratis konto.

Hjemmesiden er på engelsk, men chatbotten kan forstå og skrive dansk.

Sådan kan du bruge ChatGPT

ChatGPT bruges ved at skrive forespørgsler ("prompts") i chatten. Vi har nedenfor samlet eksempler på, hvordan ChatGPT kan anvendes i typiske arbejdsopgaver for en journalist.

Arbejdsopgave: Skrive kommentarer, klummer eller artikler.

Prompt: "Jeg arbejder på en klumme om den stigende tendens til fjernarbejde. Kan du skrive et udkast, der diskuterer fordele og ulemper ved stigende fjernarbejde?"

Arbejdsopgave: Gennemgå nyheder af lokal, national og international betydning for at fastlægge hvilke emner, der skal behandles eller få tildelt historier fra redaktionen.

Prompt: "Jeg har samlet disse avisartikler om ny teknologi på arbejdsmarkedet. Kan du opsummere artiklernes indhold og foreslå tre debatemner? [Indsæt artikler]"

Arbejdsopgave: Analysere og fortolke nyheder og information fra forskellige kilder for at videreformidle informationen.

Prompt: "Jeg har modtaget reportager fra tre forskellige kilder om en politisk begivenhed i [Land Y]. Kan du analysere reportagerne, opsummere deres vigtigste punkter og foreslå en vinkel til et indslag i en nyhedsudsendelse? [Indsæt reportager]"

Gode råd til brug af ChatGPT

- ✓ **Vær specifik:** Jo mere præcist dit spørgsmål er, desto mere nøjagtigt og relevant bliver svaret.
- ✓ **Gennemgå svar:** Tjek altid om svarene er korrekte, især ved kritiske beslutninger.
- ✓ **Bevar fortrolighed:** Del ikke personlige, fortrolige eller følsomme oplysninger i chatten.

Denne guide er udarbejdet af forskere ved Økonomisk Institut (KU) i oktober 2023. For spørgsmål, kontakt venligst aph@econ.ku.

References

1. T Eloundou, S Manning, P Mishkin, D Rock, Gpts are gpts: Labor market impact potential of llms. *Science* **384**, 1306–1308 (2024).
2. KB Hvidberg, CT Kreiner, S Stantcheva, Social Positions and Fairness Views on Inequality. *Rev. Econ. Stud.* **90**, 3083–3118 (2023).
3. D Dutz, et al., Selection in Surveys: Using Randomized Incentives to Detect and Account for Nonresponse Bias, (Working Paper), Technical report (2022).
4. F Groes, P Kircher, I Manovskii, The U-Shapes of Occupational Mobility. *The Rev. Econ. Stud.* **82**, 659–692 (2015).
5. RISJ, Reuters Institute for the Study of Journalism: What Does the Public in Six Countries Think about Generative AI in News? (https://reutersinstitute.politics.ox.ac.uk/what-does-public-six-countries-think-generative-ai-news#:~:text=Averaging%20across%20six%20countries%2C%20people,tried%20to%20use%20generative%20AI) (2024) [Accessed 28-June-2024].
6. Pew, Pew Research Center: Americans' Use of ChatGPT Is Ticking Up, but Few Trust Its Election Information (https://www.pewresearch.org/short-reads/2024/03/26/americans-use-of-chatgpt-is-ticking-up-but-few-trust-its-election-information) (2024) [Accessed 28-June-2024].
7. D Acemoglu, et al., Automation and the Workforce: A Firm-Level View From the 2019 Annual Business Survey, (National Bureau of Economic Research), Technical report (2022).
8. A Humlum, BB Meyer, *Artificial Intelligence and College Majors.* (Rockwool Foundation Research Unit), (2022).
9. K McElheran, et al., AI Adoption in America: Who, What, and Where. *J. Econ. & Manag. Strateg.* (2024).
10. D Acemoglu, D Autor, J Hazell, P Restrepo, Artificial Intelligence and Jobs: Evidence from Online Vacancies. *J. Labor Econ.* **40**, S293–S340 (2022).
11. T Babina, A Fedyk, A He, J Hodson, Artificial Intelligence, Firm Growth, and Product Innovation. *J. Financial Econ.* **151**, 103745 (2024).
12. K Bonney, et al., Tracking Firm Use of AI in Real Time: A Snapshot From the Business Trends and Outlook Survey, (National Bureau of Economic Research), Technical report (2024).
13. S Noy, W Zhang, Experimental Evidence on the Productivity Effects of Generative Artificial Intelligence. *Science* **381**, 187–192 (2023).
14. A Humlum, JR Munch, Globalization, Flexicurity and Adult Vocational Training in Denmark. *Mak. Glob. More Incl.* p. 51 (2019).
15. CR Sunstein, RH Thaler, *Nudge: Improving Decisions about Health, Wealth, and Happiness.* (Yale University Press), (2008).
16. K Feil, J Fritsch, RE Rhodes, The intention-behaviour gap in physical activity: A systematic review and meta-analysis of the action control framework. *Br. J. Sports Medicine* **57**, 1265–1271 (2023).
17. S Jäger, C Roth, N Roussille, B Schoefer, Worker Beliefs About Outside Options. *The Q. J. Econ.* p. qjae001 (2024).
18. R Gronau, Wage Comparisons–a Selectivity Bias. *J. Polit. Econ.* **82**, 1119–1143 (1974).
19. JJ Heckman, Sample Selection Bias as a Specification Error. *Econom. J. econometric society* pp. 153–161 (1979).
20. JJ Heckman, E Vytlacil, Structural Equations, Treatment Effects, and Econometric Policy Evaluation 1. *Econometrica* **73**, 669–738 (2005).
21. JJ Heckman, EJ Vytlacil, Econometric Evaluation of Social Programs, Part II: Using the Marginal Treatment Effect to Organize Alternative Econometric Estimators to Evaluate Social Programs, and to Forecast Their Effects in New Environments. *Handb. Econom.* **6**, 4875–5143 (2007).
22. P Carneiro, JJ Heckman, EJ Vytlacil, Estimating Marginal Returns to Education. *Am. Econ. Rev.* **101**, 2754–2781 (2011).
23. M Bhuller, GB Dahl, KV Løken, M Mogstad, Incarceration, Recidivism, and Employment. *J. Polit. Econ.* **128**, 1269–1324 (2020).
24. A Abadie, Semiparametric Instrumental Variable Estimation of Treatment Response Models. *J. Econom.* **113**, 231–263 (2003).
